# Machine Learning for Absolute Quantification of Unidentified Compounds in Non-Targeted LC/HRMS

**DOI:** 10.3390/molecules27031013

**Published:** 2022-02-02

**Authors:** Emma Palm, Anneli Kruve

**Affiliations:** Department of Materials and Environmental Chemistry, Stockholm University, Svante Arrhenius Väg 16, 114 18 Stockholm, Sweden; emma.palm@mmk.su.se

**Keywords:** random forest, non-target analysis, suspect screening, quantification

## Abstract

LC/ESI/HRMS is increasingly employed for monitoring chemical pollutants in water samples, with non-targeted analysis becoming more common. Unfortunately, due to the lack of analytical standards, non-targeted analysis is mostly qualitative. To remedy this, models have been developed to evaluate the response of compounds from their structure, which can then be used for quantification in non-targeted analysis. Still, these models rely on tentatively known structures while for most detected compounds, a list of structural candidates, or sometimes only exact mass and retention time are identified. In this study, a quantification approach was developed, where LC/ESI/HRMS descriptors are used for quantification of compounds even if the structure is unknown. The approach was developed based on 92 compounds analyzed in parallel in both positive and negative ESI mode with mobile phases at pH 2.7, 8.0, and 10.0. The developed approach was compared with two baseline approaches— one assuming equal response factors for all compounds and one using the response factor of the closest eluting standard. The former gave a mean prediction error of a factor of 29, while the latter gave a mean prediction error of a factor of 1300. In the machine learning-based quantification approach developed here, the corresponding prediction error was a factor of 10. Furthermore, the approach was validated by analyzing two blind samples containing 48 compounds spiked into tap water and ultrapure water. The obtained mean prediction error was lower than a factor of 6.0 for both samples. The errors were found to be comparable to approaches using structural information.

## 1. Introduction

In medicine, agriculture, and industry, thousands of chemicals are used every day, and these may end up in the environment [1]. Furthermore, many of the compounds form different transformation products through environmental photolysis, biodegradation, or disinfection [2]. To identify emerging contaminants, non-targeted analysis with liquid chromatography electrospray ionization high resolution mass spectrometry (LC/ESI/HRMS) is increasingly employed [3]. For the identification of the detected contaminants, chromatographic and mass spectrometric information is used to yield tentative candidate structures while analytical standards are required for the full identification [4]. Due to the lack of analytical standards for a majority of the tentatively identified compounds, the results of non-targeted analysis are primarily qualitative rather than quantitative [5]. Therefore, compounds whose structure cannot be unequivocally identified, or for which standards cannot be obtained, are usually overlooked when estimating the hazard [6,7]. The difficulty in quantification of the detected compounds arises from the great variations in ionization efficiency between compounds in the electrospray ionization source, which spans over six orders of magnitude [8]. Due to this difference, two compounds may give very different signals in the mass spectrometer even if the concentrations are equal [9]. For this reason, absolute quantification of the identified compounds is primarily possible using analytical standards. 

To overcome these limitations, machine learning approaches for predicting the ionization efficiency of the detected compounds have recently been developed [10,11,12,13]. The predicted ionization efficiencies can be further used to quantify the tentatively identified compounds if analytical standards are lacking [11,14,15]. These approaches rely on descriptors deduced from the structure of the compound and, therefore, at minimum a tentatively known structure of the detected compound is required. However, the concentration of a detected compound is required for risk assessment even if the confidence in the structure is low.

A different strategy has been proposed by Pieke et al. [16] which does not require structural information. In this approach, the sample is spiked with several standard compounds and the response factor of the closest eluting standard is assigned to the detected compound. This approach assumes that compounds eluting close to each other have very similar ionization efficiencies and thus do not require the structure of the detected compound. Indeed, ionization efficiency and retention time are influenced by some common factors. The retention time in reversed phase chromatography is primarily influenced by the polarity of the compound, where less polar compounds generally elute later [17]. Similarly, compounds with nonpolar moieties tend to have higher ionization efficiency as they effectively compete for the surface charge of the electrospray droplets [18,19]. Both retention time and ionization efficiency are also influenced by p*K_a_*. For example, strong bases tend to yield cations through protonation and elute faster than the respective neutral form and tend to have higher ionization efficiencies in positive ESI mode (ESI+) as protonation in the mobile phase is beneficial to ionization [20,21]. Still, many compounds that are extremely weak bases in the mobile phase, such as phthalates or esters, can be protonated in electrospray and a signal detected [8]. This can be explained as the protonation of the compounds which may already occur in the mobile phase, on the droplet surface which has a higher proton concentration than the droplet center [22] or in the gas phase. However, it has been observed that among compounds of similar polarity, stronger bases usually possess higher ionization efficiency in ESI+ [8]. Noteworthy though is that pH effect on ionization efficiency is unclear and a so called “wrong-way round” ionization may occur where the cations show highest response in basic mobile phase. Additionally, the organic modifier percentage can significantly alter the pH of the mobile phase and the p*K*_a_ of the compounds [23]. As such, the p*K*_a_ of the compound and the pH of the buffer do not alone determine whether an analyte will ionize. For example, significantly different ionization efficiencies have been observed for the same compound in different buffers with the same pH [24].

While using retention time to describe the ionization efficiency is a good start, there may be other LC/ESI/HRMS characteristics which are correlated to ionization efficiency that are still unexplored. For example, compounds need to have some acidic functionalities to become deprotonated in negative ESI mode (ESI–). Here we develop an approach that predicts the LC/ESI/HRMS response factor from the chromatographic and mass spectrometric characteristics, called the LC/MS descriptors model throughout the paper. We use the predicted response factors to quantify the compounds detected in non-target analysis without the previous structural assignment. The approach has been developed on a dataset of 92 compounds and tested on 28 compounds analyzed at three mobile phases with different pH in both positive and negative ionization mode. We further compare the performance with two baseline approaches, one assuming equal response for all compounds, and one using the response factor of the closest eluting standard. Additionally, we compare the prediction accuracy of the LC/MS descriptors model with an approach proposed previously by Liigand et al. [11] that uses the structure of the detected compounds for predicting the response. The performance of the proposed approach was validated for 48 compounds in blind spiked tap and ultrapure water samples.

## 2. Methods

### 2.1. Chemicals

Acetonitrile, methanol, and acetone (HPLC grade, Riedel-de-Haën, Seelze, Germany) were used as solvents as well as 0.1% formic acid (Merck, Darmstadt, Germany) in ultrapure water. The ultrapure water with resistance 18.2 MΩ cm and TOC < 5 ppb was prepared by Milli-Q IQ 7000 device from Merck (Darmstadt, Germany).

The aqueous mobile phase components were 0.1% formic acid solution (pH 2.7), 5.0 mM ammonium bicarbonate buffer (pH 8.0), and 0.1% ammonium solution (pH 10.0). Formic acid, ammonium bicarbonate were purchased from and ammonium (25%, MS grade) was purchased from Merck (Darmstadt, Germany). The organic phase used was acetonitrile (HPLC grade, Riedel-de-Haën, Seelze, Germany).

### 2.2. Compounds

Altogether three sets of compounds were analyzed independently. The training set contained 101 compounds out of which 92 were detected, while the test set contained 31 compounds, of which 28 were detected. The list of compounds for both sets, as well as their measured retention times and response factors, is presented in Supplementary Materials (Tables S1 and S2). Standard solutions where prepared with concentrations ranging from 1.7 × 10^−6^ to 2.4 × 10^−5^ M depending on the compound from stock solutions (approximately 1000 mg/kg, one year old). Five dilutions of each standard solution were prepared and analyzed for both the training and test set. The solution containing the training set compounds was diluted by a factor of 1, 2, 4, 20, and 40 and the solution containing test set compounds was diluted by a factor of 1, 2, 10, 20, and 100. Stock solutions used were stored at −19 °C.

### 2.3. Instrumental

Samples were analyzed with LC/ESI/HRMS in parallel in positive and negative ionization mode. Analysis was performed on a Thermo Scientific Dionex Ultimate 3000 (ThermoFisher Scientific^TM^, Waltham, MA, USA) with an RS binary pump and a Thermo Scientific Q Exactive Orbitrap (ThermoFisher Scientific^TM^, Waltham, MA, USA). A Kinetex 2.6 µm EVO C18 (150 × 3.0 mm) reversed phase column from Phenomenex (Torrence, CA, USA) was used for all LC runs. The gradient started with 5% acetonitrile and was increased to 100% over 20 min, was kept at 100% acetonitrile for 5 min, and then lowered back to 5% over 0.1 min. The system was equilibrated for 5 min between runs. The column oven temperature was 40 °C and the mobile phase flow rate was 0.350 mL/min. The autosampler temperature was kept at 15 °C. The scan range was *m*/*z* 65 to 975 Da and the resolution 120,000. Auxiliary gas, sheath gas, and sweep gas flow rates were set to 3, 35, and 0 arbitrary units, respectively. The auxiliary gas and capillary temperature were 320 °C and the S-lens RF level was 50%. 

### 2.4. Data Processing

The LC/ESI/HRMS results were extracted using Thermo Xcalibur Processing Setup Quan Identification and Thermo Xcalibur Quan Browser (Thermo Fisher Scientific^TM^, Waltham, MA, USA) and Compound Discoverer version 3.2 (Thermo Fisher Scientific^TM^, Waltham, MA, USA). [25]. For all datasets the peaks of the protonated or deprotonated ions were integrated depending on the ionization mode. The presence of sodium adducts at pH 2.7 was also checked and recorded as yes/no. For Quan Browser the mass tolerance was set to 10 ppm and peak integration was adjusted manually when necessary. For Compound Discoverer the mass tolerance was set to 5 ppm, and the maximum retention time shift was set to 1.5 min for the alignment. The S/N threshold was set to 3 and the minimum peak intensity was set to 10,000 for compound detection. Grouping was done with a mass tolerance of 5 ppm and an RT tolerance of 0.1 min. The results were then filtered based on the suspect lists and noise peaks were removed. The “Fill Gaps” and “Search Mass List” nodes were used with default settings. Some compounds yielded split peaks. In these cases, the peak areas of the two split peaks were summed and the retention time was taken as the mean retention time. The retention time in the final dataset was taken as the mean retention time over all dilutions. The slopes in the linear range of the calibration curves of the training and test data were calculated using R version 4.0 [26] and were used as the response factors in the further analysis. Here we assume that the response factors are primarily influenced by the ionization efficiency of the compounds. The log*P* and p*K*_a_ values were calculated with Chemicalize from ChemAxon (Budapest, Hungary) [27].

## 3. Model Development

### Model Training and Evaluation

Twelve descriptors were extracted from the raw data for each of the compounds and were used to train the models. These descriptors were selected based on their potential to describe either the polarity or acid-base properties of the compounds as these have been factors known to influence the ionization efficiency in ESI. The following descriptors were selected: the logarithm of the relative intensities of the peak areas in positive and negative mode for each pH, the retention time at each pH, the difference between the retention time at pH 8.0 and pH 2.7, the difference between the retention times at pH 8.0 and pH 10.0, the *m*/*z* of the compound, if the compound formed sodium adducts, if the compound was detected in negative mode, and if the *m*/*z* was odd or even. For the relative intensities the value was set to −999 when the compound was not detected in positive mode, and to 999 when it was not detected in negative mode. This was done to represent signals below the limit of detection as well as to allow these datapoints to remain in the dataset as the model cannot handle missing values. Since the models have been trained with random forest, which does not assume linear dependence between the descriptors and output criteria, any values that are consistent and fall far from the continuous range can be used to indicate compounds that could not be detected.

The LC/MS descriptor model was trained using Regularized Random Forest from RRF package. One model was trained for each combination of ionization mode and mobile phase pH, giving six random forest models in total. Each model was trained to predict the logarithm of the response factor. The caret package [28] with default settings was used to optimize the hyperparameters; the optimal values for each of the models can be found in Table S4. The importance of the descriptors in the random forest models was evaluated by permuting the variable values for each of the trees in the random forest regressor using the importance function from the RRF package [29].

The random forest models using LC/MS descriptors were also compared with two baseline models: (1) assuming equal response factor [30] and (2) using the closest eluting standard [16]. For the equal response factor baseline model, the mean response factor of the 92 standards in the training set was taken as the response factor for all compounds:

(1) $$\log\left(RF\right)=\frac{\sum_{i=1}^{n}\log(RF_{i})}{n}$$

where *n* is the number of compounds in the training set and *RF_i_* is the response factors of *i*-th compound.

In the closest eluting standard baseline model, [16] the compound was assigned the response factor of the standard in the training set with the closest retention time:

(2) $$RF=RF_{i}$$

where *RF_i_* is the response factor of the standard in the training set with the most similar retention time to the compound. The closest eluting standard approach was used for pH 2.7 positive mode only.

The LC/MS descriptors models as well as the baseline approaches were then employed to predict the response factors for the compounds in the test set. In the case of the LC/MS descriptors models and the equal response model, the response factors were converted back from logarithmic values and further used to estimate the concentrations of the compounds in the test set. Assuming an insignificant intercept of the calibration curve, the concentrations were estimated:

(3) $$c=\frac{Peak\;area}{RF_{pred}}$$

For the LC/MS descriptors approach, the six different models using LC/MS descriptors were applied yielding one predicted concentration for each ionization mode and mobile phase pH. Thus, up to six concentrations for the same compound were obtained giving a range of concentrations rather than a single value.

The accuracy of the predictions was expressed as a prediction error in folds and calculated as follows:

(4) $$error=max\;\left\{\;\;\begin{array}{c} \frac{c_{predicted}}{c_{actual}}\\ \frac{c_{actual}}{c_{predicted}} \end{array}\right.$$

The models were evaluated using the test set based on several performance metrics, namely, the mean, median, and maximum error as well as the percentage of compounds with a prediction error lower than a factor of 10. The exercise of analysis of samples and model development was repeated three times over the course of one year. The results obtained for the test set were similar in all cases. Here we report the results for the final model for which the standards were analyzed together with the validation samples and are therefore most valuable.

The code for all models and data for model training, testing, and validation can be found in https://github.com/kruvelab/ionization_efficiency_without_structure.

## 4. Validation

The LC/MS descriptors models were validated using four blind samples: ultrapure water and tap water both spiked at high and low concentration. Both ultrapure and tap water samples contained 63 compounds which were identified based on a list of the exact masses. Of the 63 compounds, 48 (listed in Table S3) were detected in at least one of the ionization modes using the same peak detection and integration method described in the “Data Processing” section. The developed models were used to estimate the response factors of the compounds and their respective concentrations according to Equation (3), and the prediction errors were calculated using Equation (4). The concentrations of the compounds in these samples were not known until after the quantification had been performed. Full lists of all compounds can be found in Tables S1–S3. 

The performance of the LC/MS descriptors model was also compared to a model previously published by Liigand et al. [14] This random forest model is based on PaDEL descriptors [31] calculated from SMILES representation of the chemical and include different molecular descriptors and fingerprints, for example, the number of nitrogen and hydrogen atoms, and presence of specific functional groups, but also topological and electronic descriptors. Additionally, mobile phase descriptors, such as pH of the water phase, viscosity, surface tension, and polarity index, are used to account for the effect of the mobile phase. The model was applied to the training, test and validation data described as the other previously approaches.

## 5. Results

### 5.1. Predicting Response Factors

The response factors were predicted for compounds detected in the training and test sets with positive and negative ionization mode for mobile phases with pH 2.7, 8.0, and 10.0, with the respective six models using LC/MS descriptors. The experimental response factors ranged from 1.7 × 10^12^ to 3.0 × 10^17^ M^−1^ in the training set and from 1.8 × 10^13^ to 2.9 × 10^17^ M^−1^ in the test set. A good correlation between the experimental response factor and the predicted response factor for all six developed models using LC/MS descriptors was observed in both the training and test sets, as can be seen in Figures S1 and S2. In the training set, the mean prediction error for the response factor was a factor of 2.6 and the median prediction error was a factor of 1.6. For the test set the corresponding errors were somewhat higher with a mean error of a factor of 10.4 and a median error of a factor of 2.7.

### 5.2. Predicting Concentration

**Figure 1:**
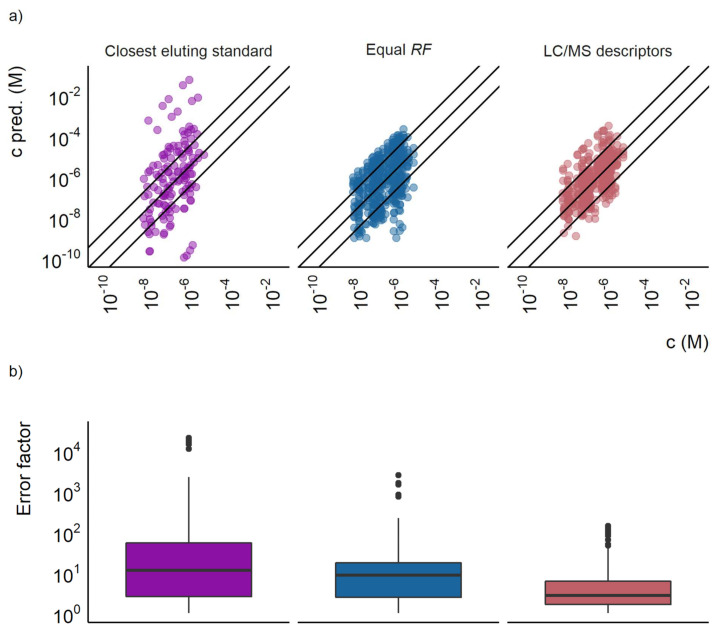
(**a**) The predicted concentration plotted against the actual concentration for the closest eluting standard approach, the equal response factor approach (equal *RF*), and the LC/MS descriptors models. Each point represents a compound at a certain concentration, the outer lines show a prediction error of a factor of 10 and the middle line show where the predicted and true concentrations are equal. (**b**) A box plot showing the prediction error of the closest eluting compound approach, the equal response factor approach, and the LC/MS descriptors model.

The predicted response factors were thereafter used to estimate the concentration of the detected compounds according to Equation (3). The concentrations in the test set ranged from 1.8 × 10^−8^ to 1.7 × 10^−5^ M. A good correlation between the predicted and spiked concentration was observed, as seen in Figure 1a. The mean prediction error for the concentrations was a factor of 3.2 for the training set and a factor of 10.2 for the test set. Median errors were a factor of 1.7 and 2.8 for the training and test set, respectively. In the training set, 98% of the datapoints (datapoints refer to compound and concentration combination in each ionization mode) had a prediction error lower than a factor of 10, the corresponding value in the test set was 83%. Therefore, concentration prediction accuracy is very close to that of the response factor and reveals that response factor prediction is the largest source of uncertainty in the predictions.

Since a different response factor prediction model was trained for each pH and ionization mode for the LC/MS descriptors models, up to six concentrations were obtained for each compound. This resulted in a range of estimated concentrations. For most compounds, this concentration range was narrower than an order of magnitude, which shows a good agreement between the models. However, for some compounds, e.g., benzotriazole and chlorothiazide, the predicted concentrations varied by over two orders of magnitude. 

The prediction accuracy varied somewhat between the six different models. In the training set the lowest mean prediction error was observed for pH 2.7 in positive mode, with a mean error of a factor of 2.2. The highest mean prediction error, a factor of 4.1, was observed for pH 10.0 in negative mode. For the test set, the lowest mean prediction error was a factor of 2.5, observed for pH 8.0 in negative mode, and the highest prediction error was a factor of 17, which was obtained for pH 2.7 in negative mode. Furthermore, at pH 2.7 negative mode, only 42% of the datapoints gave prediction errors lower than a factor of 10 for the test set, whereas for the other models this value was between 79.3% and 100%. For the test set, only 7–12 compounds could be detected in negative mode, which likely influenced the errors. All evaluation metrics for both training and test sets are given in Tables S5 and S6.

The prediction accuracy of the LC/MS descriptors models developed here was compared with the two baseline approaches. Firstly, the equal response approach for the training set resulted in a mean prediction error of a factor of 40, a median prediction error of a factor of 4.8, and 68% of the datapoints having a prediction error lower than a factor of 10. For the test set the mean error was a factor of 29, the median error a factor of 8.7, and 54% of the datapoints had prediction errors lower than a factor of 10. The distribution of over- and underpredicted concentrations was mostly even. Still, some compounds were found to have significantly underpredicted concentrations as can be seen in Figure 1a.

The second baseline approach, the closest eluting standard quantification approach proposed by Pieke et al. [16], yielded both large under- and overestimations of the concentrations. No error calculations were made for the training set for the closest eluting compound approach, since the compounds would be assigned their own response factor. However, the test set revealed large prediction errors, with a mean of a factor of 1300 and a median of a factor of 12. For this baseline approach, only 46% of the datapoints in the test set had prediction errors lower than a factor of 10. The comparison of the error distribution is visualized in Figure 1b and the agreement between the predicted and spiked concentration in Figure 1a.

### 5.3. Feature Importance

**Figure 2:**
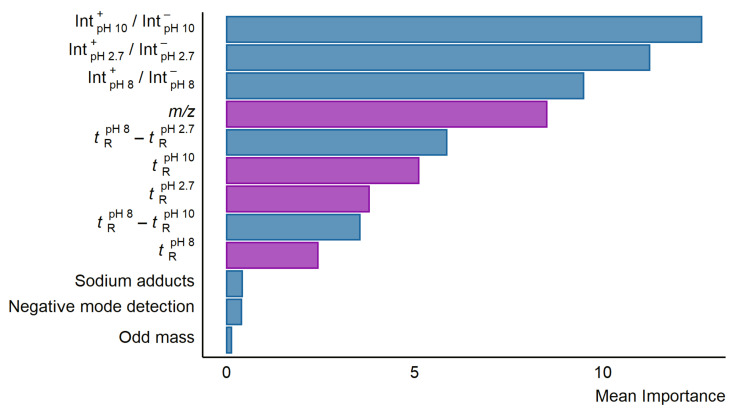
A bar plot showing the mean importance of the descriptors in the six developed random forest models using LC/MS features. The importance was calculated using the importance function from the RRF package in R. Descriptors correlated to log*P* are colored purple while descriptors in blue showed no significant correlation to neither log*P* nor p*K*_a_.

To investigate which descriptors are most useful in predicting the response factors from the LC/MS descriptors, the importance feature from RRF package was used. The three most important descriptors were the relative intensities of the peaks in positive and negative mode in the different pH mobile phases (see Figure 2). Following the intensity ratio of positive and negative mode peaks, the fourth most important feature was the *m*/*z* of the compounds. The retention time difference between pH 2.7 and 8.0 alongside other retention time descriptors were also found to be of some importance. The descriptors of least importance were formation of sodium adducts, odd-even nominal *m*/*z*, and detection in negative mode. The importance of the descriptors was mostly similar across the six models in terms of which descriptors were the most important, though the exact values differed. The importance values for all descriptors in each model can be found in Table S7.

### 5.4. Validation Results

The best performing quantification approach, i.e., the models using LC/MS descriptors, were validated with four blind samples: two ultrapure water samples and two tap water samples, spiked with 63 compounds. Depending on the mobile phase, the number of detected compounds ranged from 41 to 43 for positive detection mode, and 13 to 24 in negative detection mode, see Table S3. The concentrations of the compounds ranged from 3.8 × 10^−10^ to 1.5 × 10^−6^ M, while the predicted concentration range was between 3.6 10^−10^ and 1.3 10^−5^ M (see Figure S3). The concentration prediction accuracy was comparable for the two matrices, with the mean prediction error being a factor of 6.0 and 5.5 for the ultrapure and tap water samples, respectively. The median prediction errors were a factor of 2.6 for the spiked ultrapure water samples and a factor of 2.4 for the spiked tap water samples. Furthermore, 88% and 90% of the compounds were found to have prediction errors lower than a factor of 10 for the spiked ultrapure and tap water samples, respectively. The performance characteristics were very similar to what was found for the test set, indicating the robustness of the method.

### 5.5. Comparison with Quantification Based on Structure

**Figure 3:**
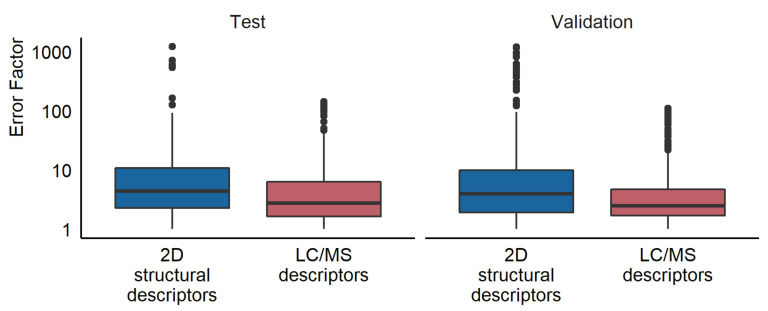
A box plot showing the prediction error for the test and validation set using the random forest LC/MS descriptors models and the 2D structural descriptors models developed by Liigand et al. [11].

Another aspect of interest was to compare the prediction accuracy of the newly developed LC/MS descriptors models to that of a model requiring structural information. For this, we used a model previously published by Liigand et al. [11] to estimate the concentrations for the compounds in both the test and the validation set. The training set was used to establish the relationship between the predicted ionization efficiency and the response factor. The mean prediction error was a factor of 16.0 for the test set, 22.9 for the validation samples in ultrapure water, and 21.0 for the validation samples in tap water (see Figure 3). The median prediction errors were a factor of 4.4 and 3.7 for ultrapure and tap water, respectively. Furthermore, 76% of the compounds had prediction errors lower than a factor of 10 in both the ultrapure and tap water samples. The results for all prediction models at each pH and ionization mode can be found in Table S8.

### 5.6. Comparison of Automatic and Manual Integration

In addition to manual peak integration, Compound Discoverer was used on the same data for the training and test sets. This was done to evaluate the effect of automatic peak integration on the quantification accuracy. The errors for the training set were found to be slightly lower for the manually integrated data with a mean error factor of 3.2 compared to 4.7 for the Compound Discoverer integrated data (see Figure S4). For the test set the difference in prediction accuracy was even larger with a mean error factor of 10.2 for the manual integration compared to 35.1 when using Compound Discoverer. 

## 6. Discussion

The prediction errors observed for the response factors in the test and training sets were very similar to the errors in the concentration prediction. This suggests that in most cases, the errors in the concentration originate from the response factor predictions rather than the concentration falling outside the linear range, or that the intercepts of the calibration curves are significant. The compounds with the largest prediction errors varied with the pH and ionization mode; however, some compounds, e.g., citrulline, had large prediction errors in several modes. 

### 6.1. Comparison to Baseline Models

The LC/MS descriptors models showed lower prediction errors compared to the baseline approaches using the equal response and the closest eluting compound approach. This is expected, especially for the equal response approach, as the response factors spanned over five orders of magnitude in both the training and test sets. This indicates a very large variability of ionization efficiency between different compounds. As can be seen in Figure 1a, the largest prediction errors in the equal response factor approach are caused by the underestimated concentrations. The over- and underestimation is likely to vary depending on the distribution of the response factors in the test and training sets. In this case, the response factors in the training set were non-normally distributed for any mode or pH. For the positive mode analysis, the distribution was skewed towards higher response factors, with many compounds having a response factor between 10^15^ and 10^17^ M^−1^ and a few compounds having response factors as low as 10^12^ M^−1^. Therefore, in positive mode, some of the largest prediction errors were seen for the compounds with lower response factors.

For the LC/MS features models some of the largest errors were also observed for the highest and lowest response factors. This is expected to occur due to “regression towards the mean”, namely, it is more beneficial for the machine learning algorithms to predict response factors close to the mean value if a clear pattern explaining the extreme values cannot be found. Therefore, interpolating models, such as random forest, often yield highest prediction errors for datapoints with most extreme values. Some examples of such compounds include carbazole and citrulline, with response factors of 6.0 × 10^12^ M^−1^ and 2.0 × 10^13^ M^−1^, respectively. In negative mode this effect was much weaker as the distribution of response factors was centered around 10^15^, although in this case, a lower number of compounds was detected.

For the closest eluting compound approach, the source of the large prediction errors can be found in the lack of a clear trend between retention time and response factor (Figure S5). For example, atraton, with a retention time of 3.5 min at pH 2.7, would indicate high polarity and therefore a low response factor, as less polar compounds are less attracted to the droplet surface. Yet, it has the fifth highest response factor in the training set. A possible reason for this is that atraton is protonated under these chromatographic conditions, resulting in high ionization efficiency despite the low polarity. There are several compounds with similar trends. This means that for these protonated compounds retention time alone is not a reliable indicator of ionization efficiency. Furthermore, even for the compounds which are neutral at given mobile phase conditions, no correlation between the response factor and the retention time could be found. This means that the compound and its closest eluting standard may have very different response factors, which results in large prediction errors. For example, diazinon, with a response factor of 1.2 × 10^17^ M^−1^ and a retention time of 13.9 min, was assigned the response factor of carbazole, which had a response of 6.0 × 10^12^ M^−1^ and a retention time of 13.6 min. This resulted in the largest prediction error for diazinon, up to a factor of 21,000 depending on the concentration. Carbazole and diazinon also have very different acid-base properties, with carbazole having a strongest acidic p*K*_a_ of 15.0 and no basic p*K*_a_, while diazinon has a strongest basic p*K*_a_ of 4.2. The log*P* values are 3.1 for carbazole and 4.2 for diazinon. These differences in physicochemical properties are likely to contribute to the significantly different response factors. 

One of the lowest prediction errors for this model was observed for ofloxacin, which had a response factor of 1.7 × 10^16^ M^−1^ and was assigned the response factor of *o*-desmethylvenlafaxinee, which had a response factor of 3.0 × 10^16^ M^−1^. Their log*P* values were somewhat different, 2.3 for *o*-desmethylvenlafaxine and 0.1 for ofloxacin. The p*K*_a_ were similar with ofloxacin having a basic p*K*_a_ of 6.7 and *o*-desmethylvenlafaxine having a basic p*K*_a_ 8.9. It would therefore appear that even compounds with rather different properties can have similar response factors and retention times. This, in combination with the lack of correlation between response factors and retention time, seen in Figure S5, makes it likely that higher prediction accuracy for some compounds results from chance alone, rather than from similar physicochemical properties.

### 6.2. Comparison between Different pH Mobile Phases

It was noticed that the prediction errors for the LC/MS descriptors models were generally lower for the mobile phase with pH 2.7 in positive mode and for mobile phase with pH 8.0 in negative. Similar findings were observed previously [11] and have been associated with the lower ionization efficiencies observed at higher pH for ESI positive mode. However, while some of the poorly predicted compounds in this study had very low response factors, some of the highest prediction errors in the test set were seen for the compounds with some of the highest response factors.

For some compounds with large prediction errors, a trend could be seen for the error factor and the pH, namely, for febantel and diazinon in the test set, the response factor was constant between different pH, but the predicted response factor decreased with the pH. This may arise from the fact that the response factors generally decreased with pH for the compounds in the training set. This led to an increased underestimation of the response factor at pH 8.0 and 10.0 in comparison to pH 2.7. On the contrary, other compounds, e.g., citrulline and dimethyl phthalate, which also had large prediction errors, did not show the same trend. 

### 6.3. Comparison with Response Factors Predicted from 2D Descriptors

Previously, ionization efficiency values have been predicted from the 2D structural descriptors of the compounds and been applied for the quantification [11]. We also compared the LC/MS descriptors approach developed in this study with the 2D structure-based predictions. The model trained in the current study, using chromatographic and mass spectrometric descriptors, showed higher prediction accuracy for both the test and validation sets on all evaluation metrics compared to the structure-based predictions. However, the current model was validated on the data collected with the same instrument as the training data, while the model by Liigand et al. [11] is a generic model transferred to our instrument. It is likely that if the models developed here were transferred to a different instrument, similar prediction errors could be expected. A previous study on the transferability of ionization efficiency scales between instruments found that the root mean square error of the predicted ionization efficiency was at most a factor of 5.2 (0.72 logarithmic units) when using a linear model with three anchoring compounds [32]. 

Interestingly, the compounds showing large prediction errors were different between the models. For example, in the test set, the three highest prediction errors were observed for diazinon, febantel, and dimethyl phthalate for the LC/MS descriptors model, whereas the 2D structural parameters models yielded the highest errors for naproxen, 5,5-diphenylhydantion, and diclofenac. This suggests that the models operate differently and can learn different information from the data. Still, it does suggest that chemical characteristics influencing ionization efficiency can be extracted from the chromatographic and mass spectrometric properties of the detected compounds, and thus, concentration estimations do not have to rely on structural knowledge alone.

### 6.4. Interpretation of the LC/MS Descriptor Model

It was of interest to understand the chemical information learned by the LC/MS descriptors models to improve the understanding of the ionization process and predictions of ionization efficiency. The most important descriptors in the model were peak area ratio between positive and negative mode at the different mobile phase pH values. Interestingly, these ratios did not yield a strong correlation to neither log*P* nor p*K*_a_ (R^2^ < 0.20). Our hypothesis is that these descriptors still give some indication of physicochemical properties relevant for ionization in ESI, see discussion in Simplifying the Model. 

For many of the developed models it seemed that more than one positive/negative mode ratio was important. Possibly, this arises as some compounds were detected in both positive and negative mode only at some specific pH. The second set of influential descriptors was the exact mass, *m*/*z*, of the detected compound. The *m*/*z* showed a linear correlation to log*P*; however, no direct correlation between the *m*/*z* and the response factor could be seen.

Retention times at different pH were also important descriptors, and it was of interest to investigate if they correlated to the polarity and acid-base properties of the compounds. Not surprisingly, the retention time at all pHs correlated to log*P*. Another interesting aspect was to investigate if the compounds which became protonated in the acidic mobile phase could be pinpointed based on the retention time differences from one pH to another, namely, weakly basic compounds should yield a shift in retention time when the mobile phase pH is increased, and the compounds become neutral. Compounds which showed a shift to longer retention times at pH 10.0 generally had higher response factors in positive mode, indicating that these compounds may be basic. Similarly, compounds which shifted to shorter retention times generally had lower response factors in positive mode, indicating that these may be acidic. This supports the hypothesis that compounds that are already ionized in the mobile phase are also easier to be ionized in ESI, which has previously also been suggested by Kruve et al. [33]. However, no clear correlation could be found between the shift in the retention time and p*K*_a_ of the compounds. It is also worth noting that the p*K*_a_ values used were calculated in pure water and the mobile phase pH values were measured in the water phase; however, the p*K*_a_ and pH values in the buffer and acetonitrile mixture used in chromatographic analysis is likely to be somewhat different [34].

The three least important descriptors for the LC/MS descriptors models were detection in negative mode, sodium adduct formation, and if the mass was odd or even. In the case of the negative mode detection, this is expected as this information is already explained by the peak area ratios. For the sodium adduct formation, the hypothesis was that compounds which form sodium adducts often are oxygen bases, i.e., weaker bases, and may thus have a lower response factor. However, no correlation between the formation of sodium adducts and the response factor of the compound alone was observed. The odd or even mass would indicate if the number of nitrogen atoms is odd or even. The presence of nitrogen atoms makes the compound more likely to be basic, and thus an odd mass could imply that the compound is a base. However, compounds with an even number of nitrogen atoms and compounds with no nitrogen atoms are also grouped together by this feature. 

**Figure 4:**
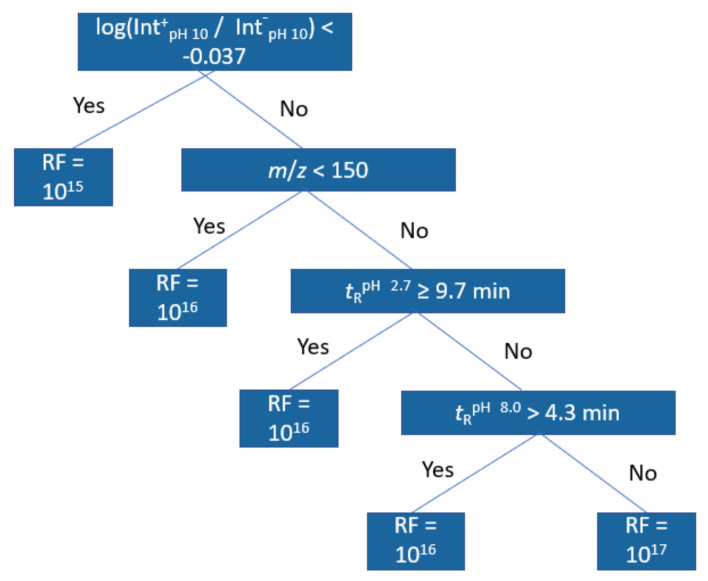
Regression tree trained from the training set data at pH 2.7 positive mode.

For further interpretation of the models, regression trees were trained with the same dataset; Figure 4 showing an example of this at pH 2.7 positive ionization mode. The first node in all positive mode trees was the positive/negative mode peak area ratio at pH 10.0 indicating a lower response factor for compounds with lower ratio. The second and third node for positive mode in pH 2.7 and 8.0 related to retention time at pH 2.7 and the molecular weight of the compound, with compounds of higher *m*/*z* and compounds with longer retention time both having higher response factors. The fourth and fifth nodes were again related to the retention times and the peak area ratios, but at pH 8.0. For negative mode models, the trees were shallower, but still the main important descriptors were found to be the *m*/*z* and the peak area ration in positive and negative mode. In this case, compounds with higher peak area ratios were predicted to have lower response factors and compounds with higher *m*/*z* were predicted to have higher response factors. All regression trees can be found in Figures S6–S11.

### 6.5. Simplifying the Model

For the compounds which had peaks detected in both positive and negative mode, a correlation between the logarithm of the response factor and the intensity ratio was observed at all mobile phase pHs with R^2^ values between 0.48 and 0.75, as can be seen in Figure S12. In positive mode, compounds with higher ratios tended to have higher response factors whereas in negative mode, compounds with lower ratios tended to have higher response factors. 

**Figure 5:**
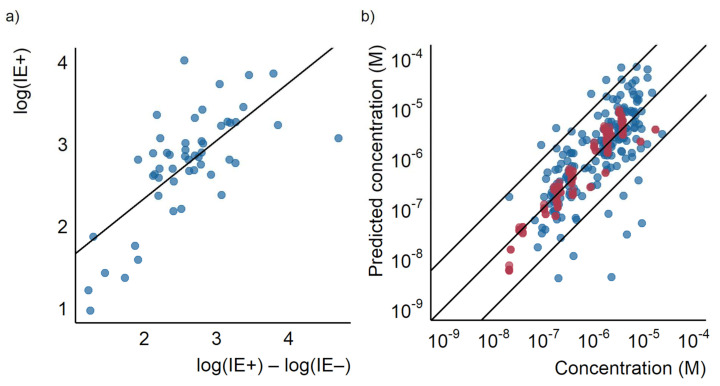
(**a**) A scatterplot showing the difference between the logarithm of the ionization efficiencies ion positive and negative modes measured by Liigand et al. [35] plotted against the logarithm of the ionization efficiency ion positive mode as well as the regression line. (**b**) The predicted concentration for the test (red) and training (blue) set based on the linear model using only the ratio of the logarithmic response factors plotted against the known concentration.

To evaluate the universality of this finding, the previously published ionization efficiencies values by Liigand et al. [35] for compounds ionizing in both positive and negative mode were analyzed. The difference in logarithmic ionization efficiency in ESI positive and negative mode was correlated with the ionization efficiencies measured in both modes. A weak correlation was observed with the R^2^ of 0.51, as can be seen in Figure 5a. Compounds which had a higher difference between the logarithmic ionization efficiency in positive mode and negative mode, also generally had higher ionization efficiencies in positive mode. This suggests that ionization in both positive and negative ionization mode is driven by the same physicochemical properties as the difference in two highly correlated variables correlates to both variables, as long as the slope of the correlation is significantly different from one. The beauty of this finding is that the physicochemical property driving the ionization process in both modes does not need to be pinpointed to make predictions about the ionization efficiency.

Therefore, for compounds ionizing in both modes, an even simpler, linear model could be used for evaluating the response factor. To investigate this further we fitted a linear regression between the logarithm of the response factor in positive mode at pH 8.0 with the logarithm of the peak area ratio in positive and negative mode at the same pH in the training set (Figure S12). The linear model was then applied to quantify the compounds detected in both modes at the same pH in the test set. A good correlation between measured and predicted concentrations for both the training and test sets was observed (see Figure 5b). The obtained mean error was a factor of 1.7 and the median error was a factor of 1.4. The maximum error was a factor of 4.7. Using this simplified model would significantly reduce the number of measurements required; however, it also excludes any compound not detected in both modes. 

### 6.6. Uncertainty Assessment

It needs to be acknowledged that the prediction accuracy of the LC/MS descriptors model is not competing with the targeted analysis. Still, the predictions can prove useful in environmental monitoring where considering the potential risk, that is hazard and exposure, of the detected compounds is important in further prioritization [36]. The uncertainty of the risk estimation needs to incorporate uncertainty from both quantification (exposure) and toxicity endpoint predictions (hazard). It is therefore of interest to compare the prediction errors for the developed approach to the errors of measured and predicted toxicities. Based on a review of the data in the CompTox [37] database, the measured toxicity endpoints (e.g., LC_50_ values) for the same compound and species tend to fall within a factor of 10, though the range can in some cases be as high as a factor of 1000. Similarly, Castro [38] found that the reported EC_50_ values for chlorinated paraffins may differ by a factor of three. For the predicted toxicity, Chen et al. [39] achieved prediction errors below a factor of 10 for most of their compounds. However, in this case errors over three orders of magnitude were also observed. These error ranges are very similar to what we observe for the predicted concentrations with the LC/MS descriptors model. In the validation set, 90% of the compounds have prediction errors lower than a factor of 10, and the maximum prediction errors fell within a factor of 1000. According to the law of propagation of error, the larger uncertainty is always dominant. For example, combining toxicity prediction and concentration prediction with an uncertainty of an order of magnitude result in a total uncertainty of a factor of 14 for the risk combining concentration and toxicity. At the same time, combining toxicity prediction with an uncertainty of an order of magnitude and concentration prediction of a factor of 1.2 (uncertainty 20%, corresponding to standard targeted analysis), yields total uncertainty of a factor of 10. Please see Supplementary Materials for more details. This suggests that the quantification accuracy achieved here, though higher then observed in targeted analysis, can be combined with toxicity predictions without dramatically increasing the uncertainty in the total risk. 

### 6.7. Limitations and Moving Forward

The application area for all models depends on the scope of data on which the model has been trained. In the case of the LC/MS descriptors models developed here, the predictions are limited to protonated [M + H]^+^ or deprotonated [M − H]^−^ molecules. The models were primarily trained on pesticides, pharmaceuticals, and other small organic molecules. The log*P* ranged from −3.9 to 6.4 and the strongest basic and acidic p*K*_a_ (for the compounds for which one could be calculated) ranged from −1.8 to 15.0 and −0.8 to 15.7, respectively.

Additionally, as response factors can vary between different instruments, the model in its current form can be applied to compounds analyzed on the same instrument. Many of the descriptors are also instrument and method specific, e.g., retention times and response factor ratios. This means that for the model to be applicable for another instrument, the training set needs to be reanalyzed on that specific instrument, and the model retrained. However, it has been found that ionization efficiencies are correlated between different instruments [40]. This has previously enabled training a single ionization efficiency model and transferring the predictions to different instruments via correlation [15]. Therefore, the transferability of the model can be further investigated in the future, alongside validation engaging several instruments and laboratories. It is expected that somewhat larger prediction errors would emerge.

The matrix of the sample, as well as any sample preparation, is another important consideration. In the current study, the model was applied to spiked water samples which were injected directly into the LC. However, more complex matrices may cause more pronounced matrix effects in terms of ionization suppression or enhancement which may increase the prediction errors [41]. Additionally, imperfect recoveries during the sample preparation can further increase quantification errors. Thus, more research into the recoveries for different sample preparation methods are needed.

As the relative peak areas were found to be the most important descriptor for the random forest models the peak integration is very important for the prediction accuracy. This was clearly seen when comparing the results from the models trained with data from completely automatic integration with Compound Discoverer to those done with manually adjusted integration in Xcalibur. The models trained and tested using data integrated with Compound Discoverer gave a mean error almost four times higher than the models trained using the manually integrated data. This difference may be explained by the way peaks are integrated in Compound Discoverer. The software fits a Gaussian function to the datapoints on the peak which can significantly change the area if the peaks are not symmetrical. This somewhat worsens both the quality of the training and test data, which in turn reduces the prediction accuracy as can be seen in Figure S4. However, the quality of peak integration affects all the machine learning models and therefore further developments in peak picking, grouping, and integration are highly needed to improve the quality of quantitative non-targeted analysis [42].

Despite the limitations, it is clear that machine learning models generally outperform the baseline models; therefore, applying machine learning models in peak prioritization and risk assessment is recommended. As the LC/MS and structure-based approaches show similar prediction accuracies there is no one recommendation for which model to use. The structure-based approach has been developed on a wider set of chemicals and instruments whereas the LC/MS based approach is applicable also to unidentified compounds. Thus, these approaches are seen as complementing each other. We recommend using structure-based predictions where candidate structures with high confidence have been assigned to the peaks, as it requires fewer measurements and is readily transferable between different instruments. In cases where no structural candidate has been assigned or confidence in the assignment is low (majority of the peaks in non-targeted LC/HRMS analysis), LC/MS feature-based predictions are recommended. The LC/MS approach may be useful for peak prioritization of peaks with yet unknown identity. In parallel, further developments in models not requiring structural assignment are the way forward in non-targeted LC/HRMS and additional developments can improve the chemical scope as well as transferability between instruments.

## 7. Conclusions

Here, we have presented a quantification strategy applicable at any level of structural identification in LC/ESI/HRMS non-targeted analysis. This approach allows for concentration estimations even if the structure of the compound is unknown, or if analytical standards are unavailable. The descriptors which were identified as most important were the ratios between peak areas in positive and negative mode, followed by the *m*/*z* of the compounds, and the difference in retention time between mobile phases with pH 2.7 and pH 8.0. The validation of the developed model gave mean prediction errors with factor of 6.0 and gave comparable prediction errors to a previously developed model which uses structural parameters as descriptors. However, the model was validated on the same instrument as it was trained on, and as such, any transference of the model to a different instrument would require some retraining and could likely cause somewhat higher prediction errors.

## Data Availability

The data is available from https://github.com/kruvelab/ionization_efficiency_without_structure.

## Supplementary Materials

The following supporting information can be downloaded, Figure S1: Predicted an measured response factors in the training set for the LC/MS features models, Figure S2: Predicted an measured response factors in the test set for the LC/MS features models, Figure S3: Predicted and concentrations for the validation set, Figure S4: Error factor distribution for the different peak integration methods, Figure S5: retention time and response factor relationship, Figures S6–S11: Regression trees, Figure S12: Response factor and peak area ratios relationships, Tables S1–S3: Compound information, Table S4: Hyper parameters, Tables S5–S6: Evaluation parameters for training and test sets, Table S7: Feature importance, Table S8: Evaluation parameters for the validation set.

## References

[1] Wang Z., Walker G.W., Muir D.C.G., Nagatani-Yoshida K. (2020). Toward a Global Understanding of Chemical Pollution: A First Comprehensive Analysis of National and Regional Chemical Inventories. Environ. Sci. Technol..

[2] Li X.-F., Mitch W.A. (2018). Drinking Water Disinfection Byproducts (DBPs) and Human Health Effects: Multidisciplinary Challenges and Opportunities. Environ. Sci. Technol..

[3] Kruve A. (2019). Semi-quantitative Non-target Analysis of Water with Liquid Chromatography/High-resolution Mass Spectrometry: How Far Are We?. Rapid Commun. Mass Spectrom..

[4] Schymanski E.L., Jeon J., Gulde R., Fenner K., Ruff M., Singer H.P., Hollender J. (2014). Identifying Small Molecules via High Resolution Mass Spectrometry: Communicating Confidence. Environ. Sci. Technol..

[5] Sobus J.R., Wambaugh J.F., Isaacs K.K., Williams A.J., McEachran A.D., Richard A.M., Grulke C.M., Ulrich E.M., Rager J.E., Strynar M.J. (2018). Integrating Tools for Non-Targeted Analysis Research and Chemical Safety Evaluations at the US EPA. J. Expo. Sci. Environ. Epidemiol..

[6] Rager J.E., Strynar M.J., Liang S., McMahen R.L., Richard A.M., Grulke C.M., Wambaugh J.F., Isaacs K.K., Judson R., Williams A.J. (2016). Linking High Resolution Mass Spectrometry Data with Exposure and Toxicity Forecasts to Advance High-Throughput Environmental Monitoring. Environ. Int..

[7] Sørensen L., McCormack P., Altin D., Robson W.J., Booth A.M., Faksness L.-G., Rowland S.J., Størseth T.R. (2019). Establishing a Link between Composition and Toxicity of Offshore Produced Waters Using Comprehensive Analysis Techniques—A Way Forward for Discharge Monitoring?. Sci. Total Environ..

[8] Oss M., Kruve A., Herodes K., Leito I. (2010). Electrospray Ionization Efficiency Scale of Organic Compounds. Anal. Chem..

[9] Cech N.B., Enke C.G. (2001). Practical Implications of Some Recent Studies in Electrospray Ionization Fundamentals. Mass Spectrom. Rev..

[10] Chalcraft K.R., Lee R., Mills C., Britz-McKibbin P. (2009). Virtual Quantification of Metabolites by Capillary Electrophoresis-Electrospray Ionization-Mass Spectrometry: Predicting Ionization Efficiency Without Chemical Standards. Anal. Chem..

[11] Liigand J., Wang T., Kellogg J., Smedsgaard J., Cech N., Kruve A. (2020). Quantification for Non-Targeted LC/MS Screening without Standard Substances. Sci. Rep..

[12] Panagopoulos Abrahamsson D., Park J.-S., Singh R.R., Sirota M., Woodruff T.J. (2020). Applications of Machine Learning to In Silico Quantification of Chemicals without Analytical Standards. J. Chem. Inf. Model..

[13] Mayhew A.W., Topping D.O., Hamilton J.F. (2020). New Approach Combining Molecular Fingerprints and Machine Learning to Estimate Relative Ionization Efficiency in Electrospray Ionization. ACS Omega.

[14] Wang T., Liigand J., Frandsen H.L., Smedsgaard J., Kruve A. (2020). Standard Substances Free Quantification Makes LC/ESI/MS Non-Targeted Screening of Pesticides in Cereals Comparable between Labs. Food Chem..

[15] Kruve A., Kiefer K., Hollender J. (2021). Benchmarking of the Quantification Approaches for the Non-Targeted Screening of Micropollutants and Their Transformation Products in Groundwater. Anal. Bioanal. Chem..

[16] Pieke E.N., Granby K., Trier X., Smedsgaard J. (2017). A Framework to Estimate Concentrations of Potentially Unknown Substances by Semi-Quantification in Liquid Chromatography Electrospray Ionization Mass Spectrometry. Anal. Chim. Acta.

[17] Jandera P. (1988). Mechanism and Prediction of Retention of Oligomers in Normal-Phase and Reversed-Phase HPLC. Chromatographia.

[18] Canals I., Portal J.A., Rosés M., Bosch E. (2002). Retention of Ionizable Compounds on HPLC. Modelling Retention in Reversed-Phase Liquid Chromatography as a Function of PH and Solvent Composition with Methanol-Water Mobile Phases. Chromatographia.

[19] Cech N.B., Enke C.G. (2000). Relating Electrospray Ionization Response to Nonpolar Character of Small Peptides. Anal. Chem..

[20] Ehrmann B.M., Henriksen T., Cech N.B. (2008). Relative Importance of Basicity in the Gas Phase and in Solution for Determining Selectivity in Electrospray Ionization Mass Spectrometry. J. Am. Soc. Mass Spectrom..

[21] Liigand J., Kruve A., Leito I., Girod M., Antoine R. (2014). Effect of Mobile Phase on Electrospray Ionization Efficiency. J. Am. Soc. Mass Spectrom..

[22] Malevanets A., Consta S. (2013). Variation of Droplet Acidity during Evaporation. J. Chem. Phys..

[23] Heller S.T., Silverstein T.P. (2020). PKa Values in the Undergraduate Curriculum: Introducing PKa Values Measured in DMSO to Illustrate Solvent Effects. ChemTexts.

[24] Ojakivi M., Liigand J., Kruve A. (2018). Modifying the Acidity of Charged Droplets. ChemistrySelect.

[25] Thermo Scientific™ (2011). Xcalibur™ Software.

[26] R Core Team (2021). R: A Language and Environment for Statistical Computing.

[27] Chemicalize Was Used for Prediction of logP and pKa. https://chemicalize.com/.

[28] Kuhn M. (2021). Caret: Classification and Regression Training.

[29] Importance Function—RDocumentation. https://www.rdocumentation.org/packages/randomForest/versions/4.6-14/topics/importance.

[30] Tang T., Zhang P., Li S., Xu D., Li W., Tian Y., Jiao Y., Zhang Z., Xu F. (2021). Absolute Quantification of Acylcarnitines Using Integrated Tmt-PP Derivatization-Based LC–MS/MS and Quantitative Analysis of Multi-Components by a Single Marker Strategy. Anal. Chem..

[31] Yap C.W. (2011). PaDEL-Descriptor: An Open Source Software to Calculate Molecular Descriptors and Fingerprints. J. Comput. Chem..

[32] Liigand J., Kruve A., Liigand P., Laaniste A., Girod M., Antoine R., Leito I. (2015). Transferability of the Electrospray Ionization Efficiency Scale between Different Instruments. J. Am. Soc. Mass Spectrom..

[33] Kruve A., Kaupmees K., Liigand J., Leito I. (2014). Negative Electrospray Ionization via Deprotonation: Predicting the Ionization Efficiency. Anal. Chem..

[34] Rosés M. (2004). Determination of the PH of Binary Mobile Phases for Reversed-Phase Liquid Chromatography. J. Chromatogr. A.

[35] Liigand P., Kaupmees K., Haav K., Liigand J., Leito I., Girod M., Antoine R., Kruve A. (2017). Think Negative: Finding the Best Electrospray Ionization/MS Mode for Your Analyte. Anal. Chem..

[36] Been F., Kruve A., Vughs D., Meekel N., Reus A., Zwartsen A., Wessel A., Fischer A., ter Laak T., Brunner A.M. (2021). Risk-Based Prioritization of Suspects Detected in Riverine Water Using Complementary Chromatographic Techniques. Water Res..

[37] Williams A.J., Grulke C.M., Edwards J., McEachran A.D., Mansouri K., Baker N.C., Patlewicz G., Shah I., Wambaugh J.F., Judson R.S. (2017). The CompTox Chemistry Dashboard: A Community Data Resource for Environmental Chemistry. J. Cheminform..

[38] Castro M., Breitholtz M., Sobek A., Gorokhova E., Asplund L., Scheringer M., Fakulteten N. (2020). Chlorinated Paraffins: Improved Understanding of Their Bioaccumulation and Toxicity in *Daphnia magna*.

[39] Chen X., Dang L., Yang H., Huang X., Yu X. (2020). Machine Learning-Based Prediction of Toxicity of Organic Compounds towards Fathead Minnow. RSC Adv..

[40] Liigand J., de Vries R., Cuyckens F. (2019). Optimization of Flow Splitting and Make-up Flow Conditions in Liquid Chromatography/Electrospray Ionization Mass Spectrometry. Rapid Commun. Mass Spectrom..

[41] Taylor P.J. (2005). Matrix Effects: The Achilles Heel of Quantitative High-Performance Liquid Chromatography–Electrospray–Tandem Mass Spectrometry. Clin. Biochem..

[42] Guo J., Shen S., Xing S., Chen Y., Chen F., Porter E.M., Yu H., Huan T. (2021). EVA: Evaluation of Metabolic Feature Fidelity Using a Deep Learning Model Trained With Over 25000 Extracted Ion Chromatograms. Anal. Chem..

