# Supplementary material for: Machine Learning for Absolute Quantification of Unidentified Compounds in Non-Targeted LC/HRMS
Source: Molecules. 2022 Feb 2;27(3):1013. doi: 10.3390/molecules27031013 (PMC8840743; doi:10.3390/molecules27031013)
Supplement: Supplementary file 1 [file molecules-27-01013-s001.zip › molecules-1552275-supplementary.pdf]

Supporting information

Machine Learning for Absolute Quantification of Unidentified Compounds in Non-Targeted LC/HRMS

Emma Palm, Anneli Kruve

Department of Materials and Environmental Chemistry, Stockholm University, Svante Arrhenius väg 16, 114 18 Stockholm, Sweden; emma.palm@mmk.su.se (E.P.); anneli.kruve@su.se (A.K.)

Table of contents

Figure S1 ····· 3

Figure S2 ····· 3

Figure S4 ····· 5

Figure S5 ····· 7

Figure S6 ····· 8

Figure S7 ····· 9

Figure S8 ····· 10

Figure S9 ····· 11

Figure S10 ····· 12

Figure S11 ····· 13

Table S1 ····· 14

Table S2 ····· 18

Table S3 ····· 20

Table S4 ····· 23

Table S5 ····· 23

Table S6 ····· 24

Table S7 ····· 25

Table S8 ····· 26

Error propagation ····· 27

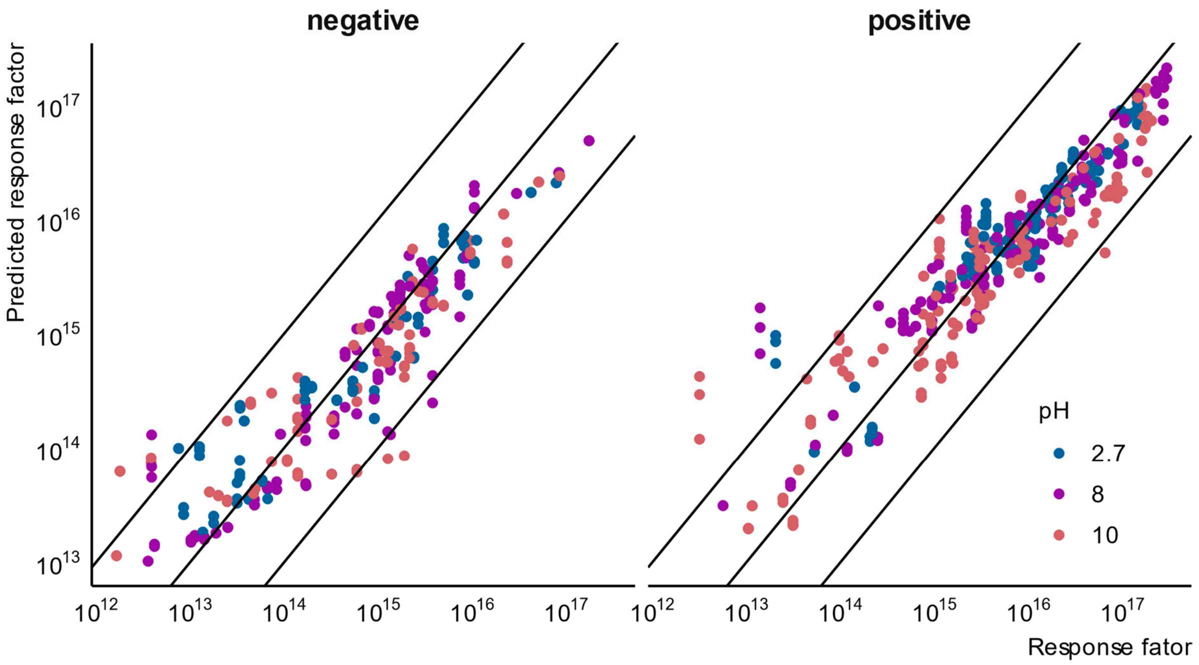

Figure S1. A scatter plot sowing the predicted and measured response factors for the LC/MS features model for all compounds at each pH and ionization mode for the training set.

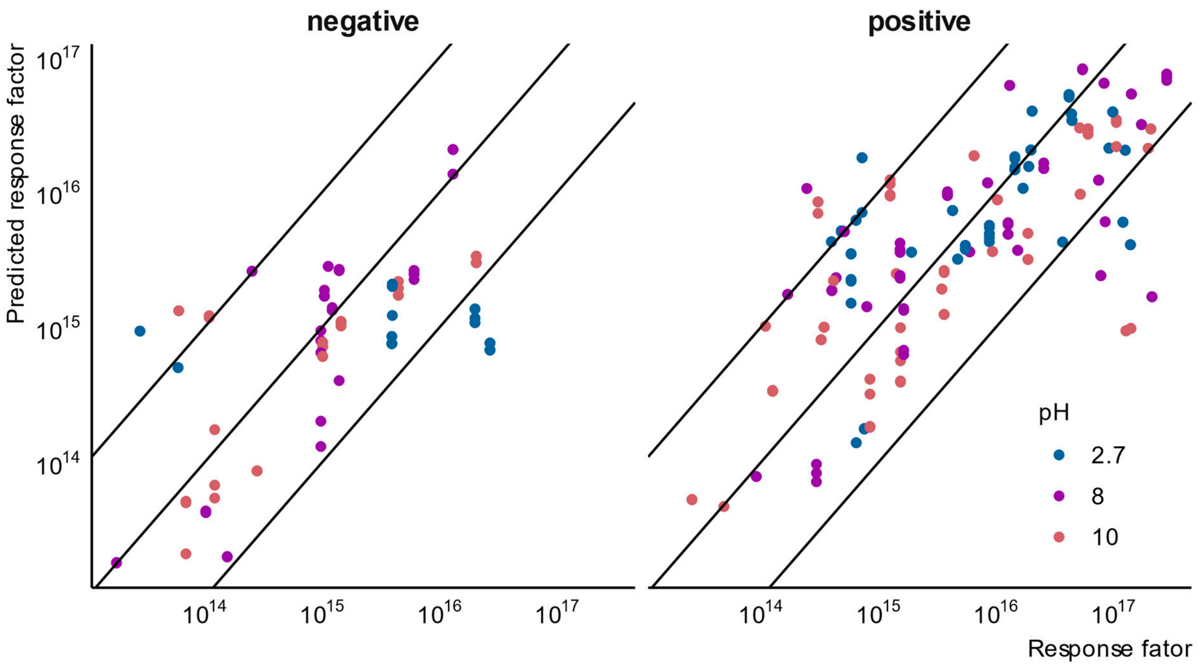

Figure S2. A scatter plot sowing the predicted and measured response factors for the LC/MS features model for all compounds at each pH and ionization mode for the test set.

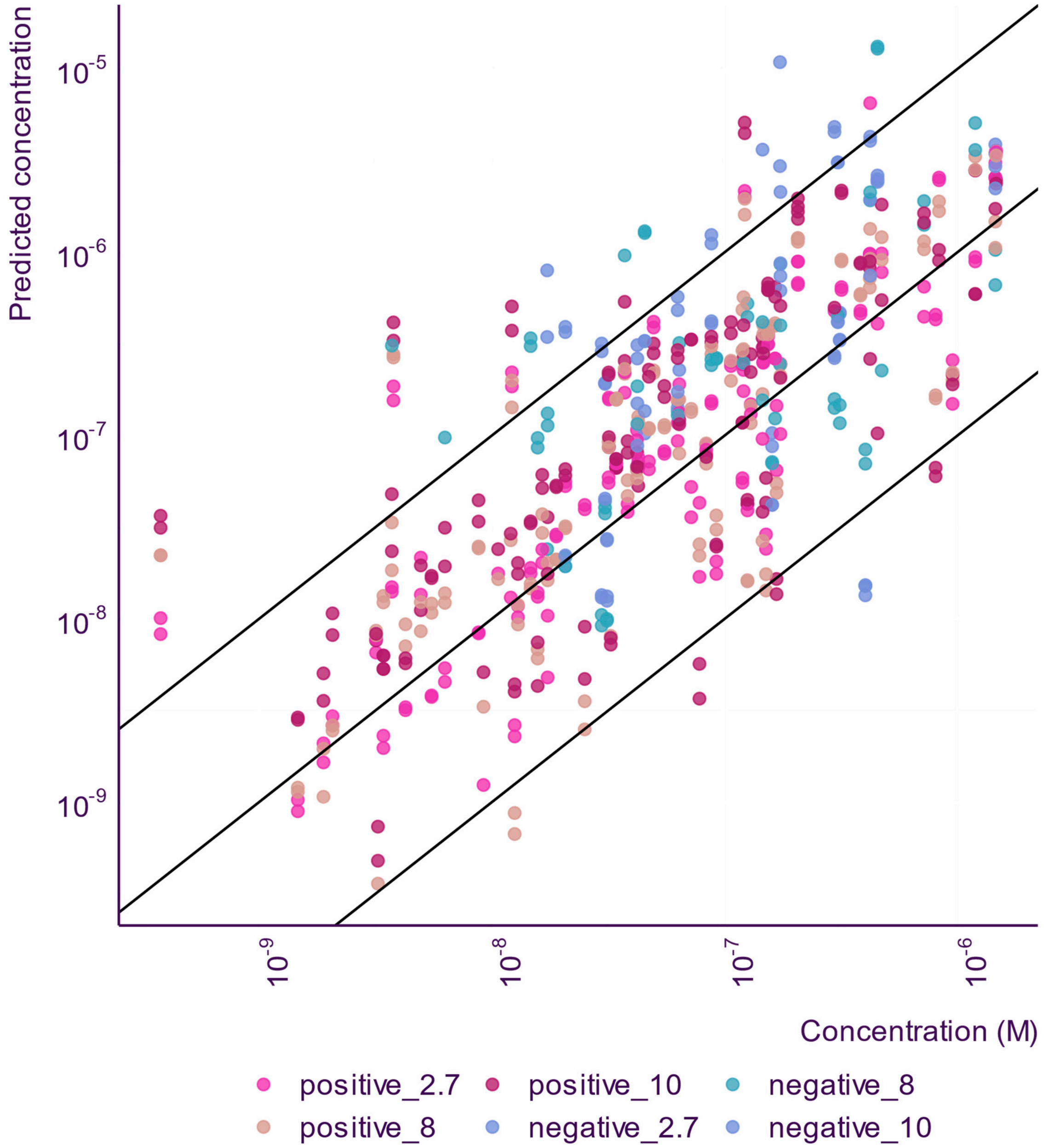

Figure S3. A scatter plot sowing the predicted and measured concentrations for the LC/MS features model for all compounds at each pH and ionization mode for the validation set.

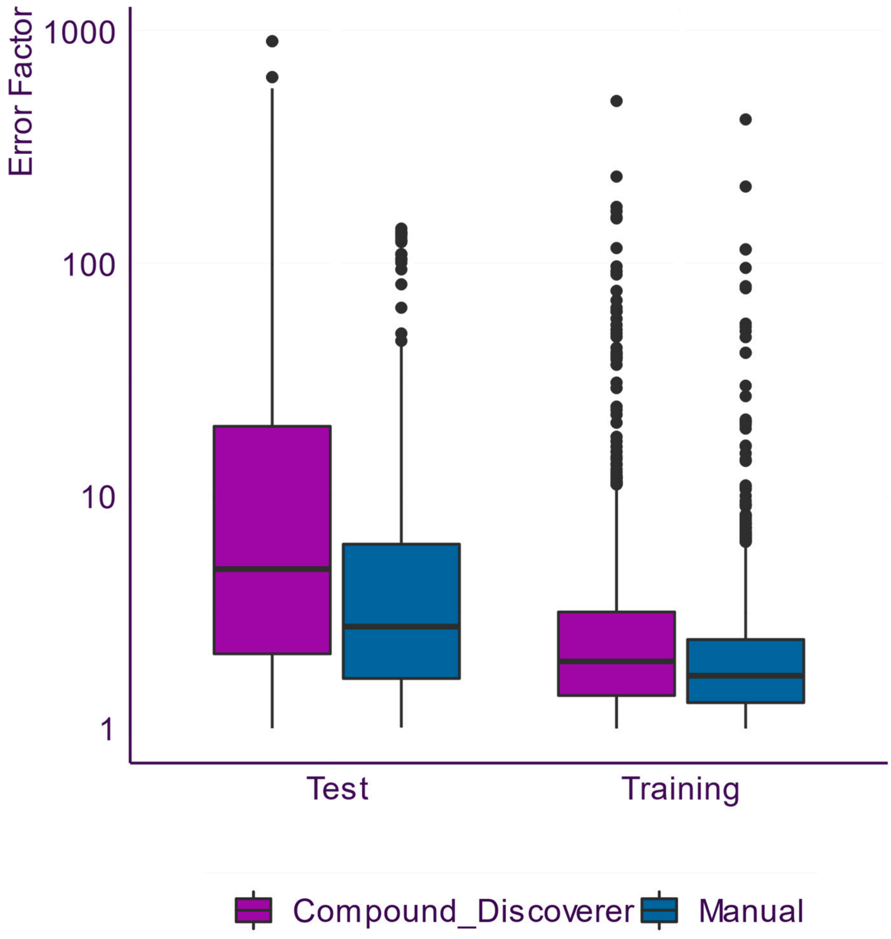

A box plot whowing the error factorfor the training and test set using datasets obtained with Manual and Compound Discoverer integrated data respectively.

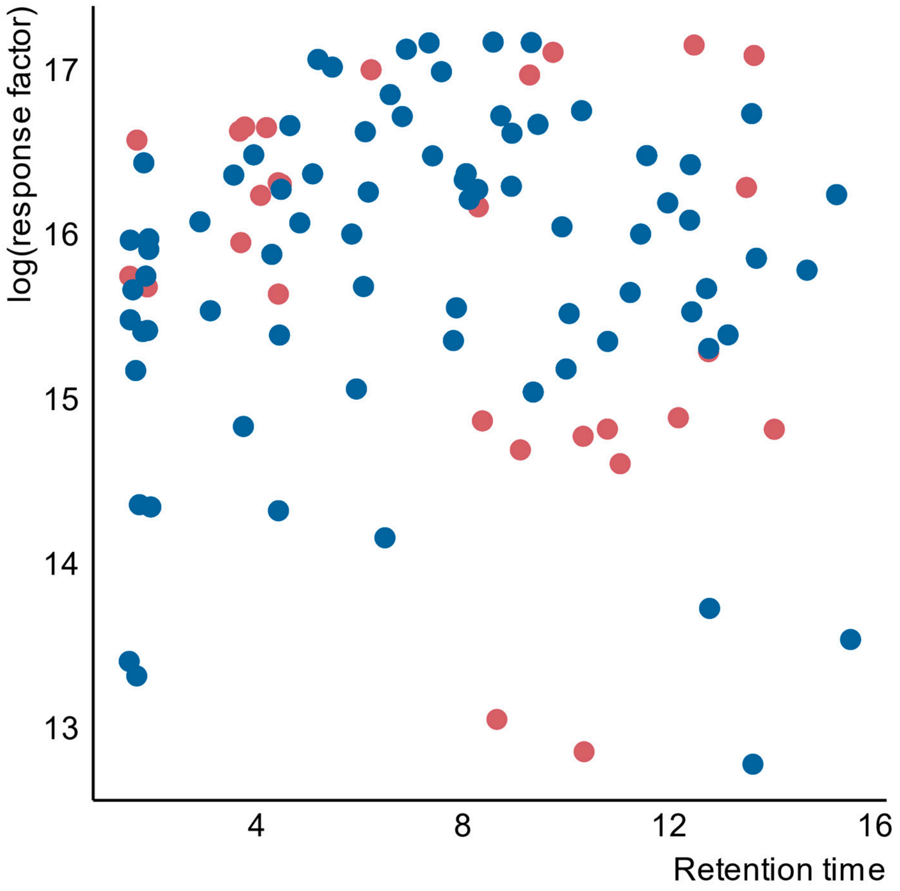

Figure S4. The scatterplot shows the logarithm of the response factor plotted against the t_R (min) for the training (blue) and test (red) set.

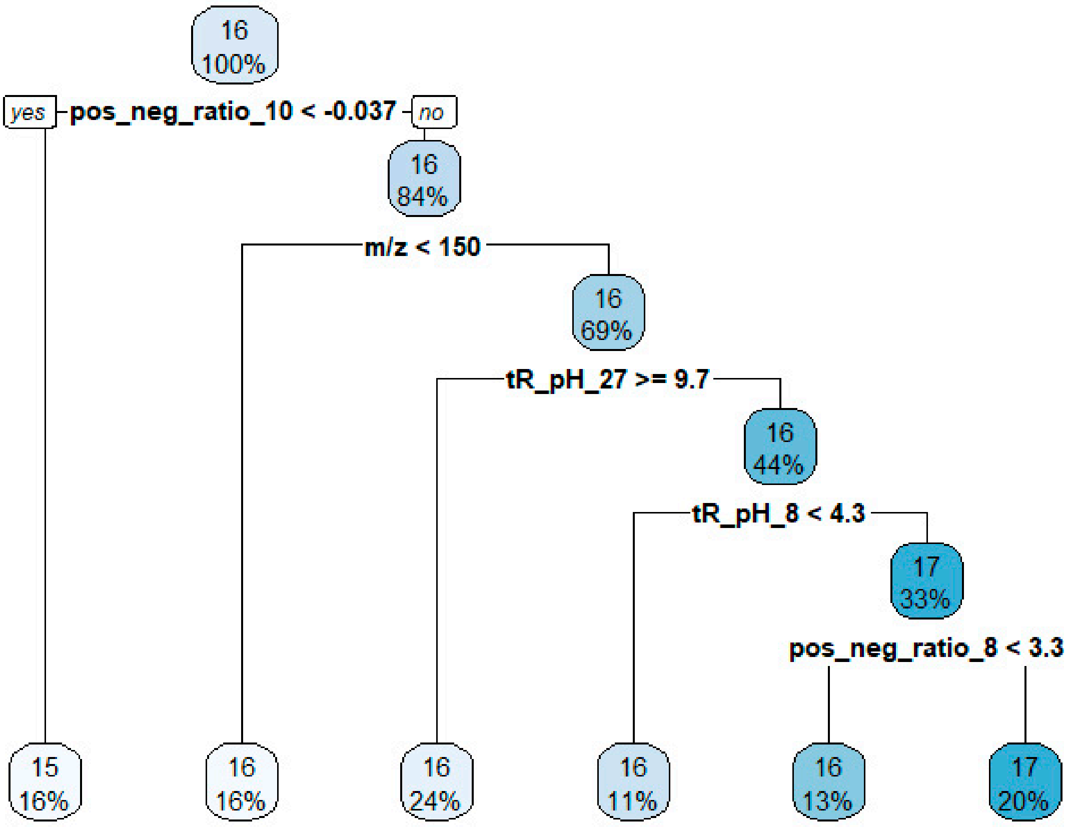

Figure S5. Regression tree trained from the training set data at pH 2.7 positive mode. In the nodes the logarithm of the response factor is found as well as the percentage of compounds belonging to that node.

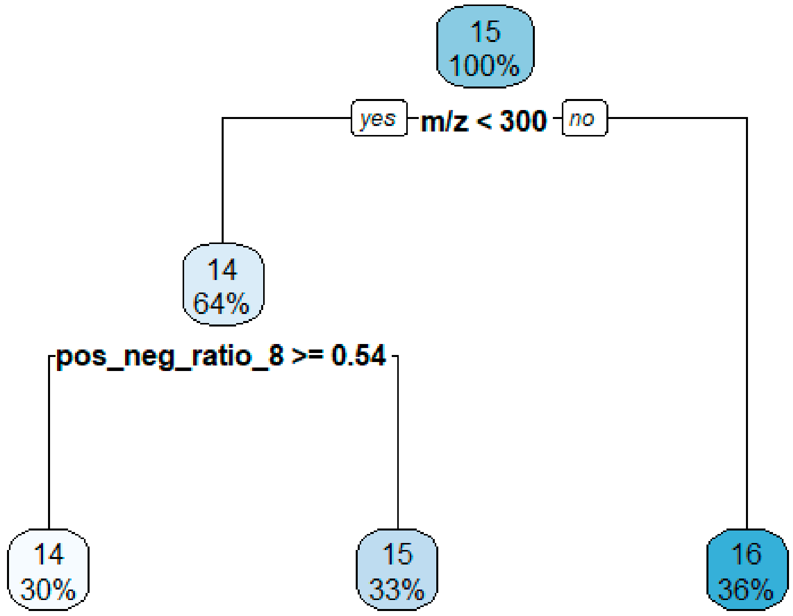

Figure S6. Regression tree trained from the training set data at pH 2.7 negative mode. In the nodes the logarithm of the response factor is found as well as the percentage of compounds belonging to that node.

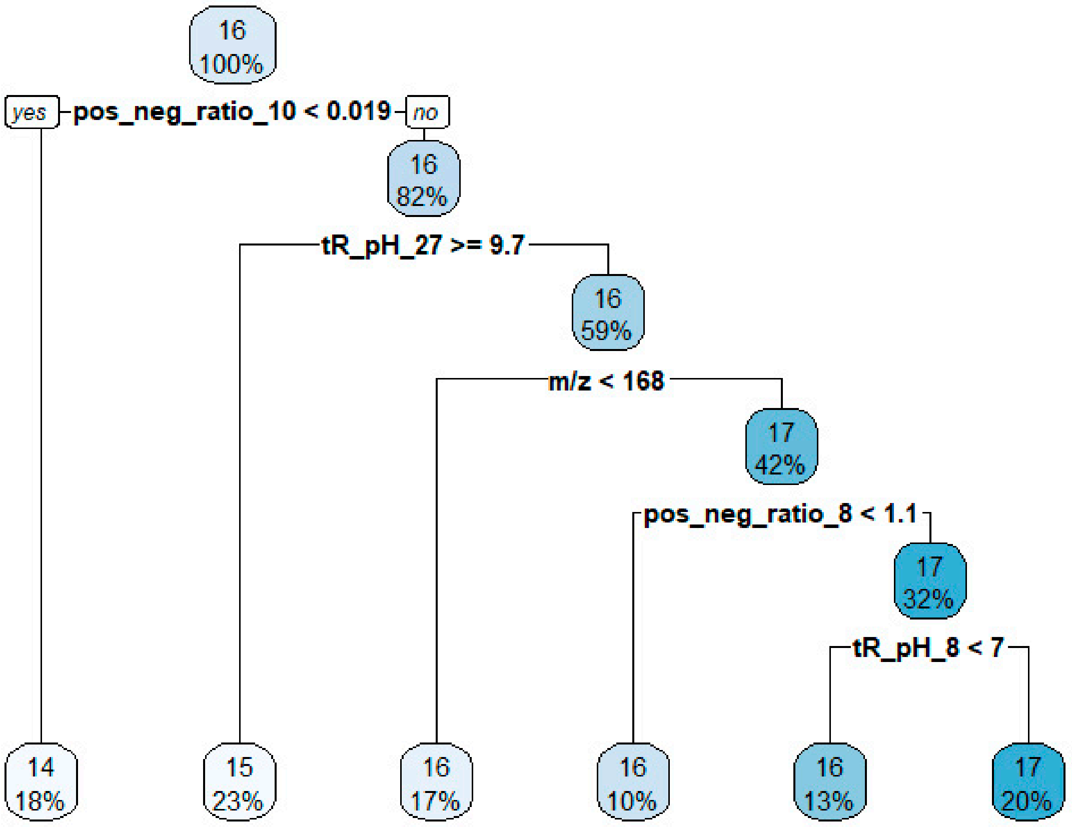

Figure S7. Regression tree trained from the training set data at pH 8.0 positive mode. In the nodes the logarithm of the response factor is found as well as the percentage of compounds belonging to that node.

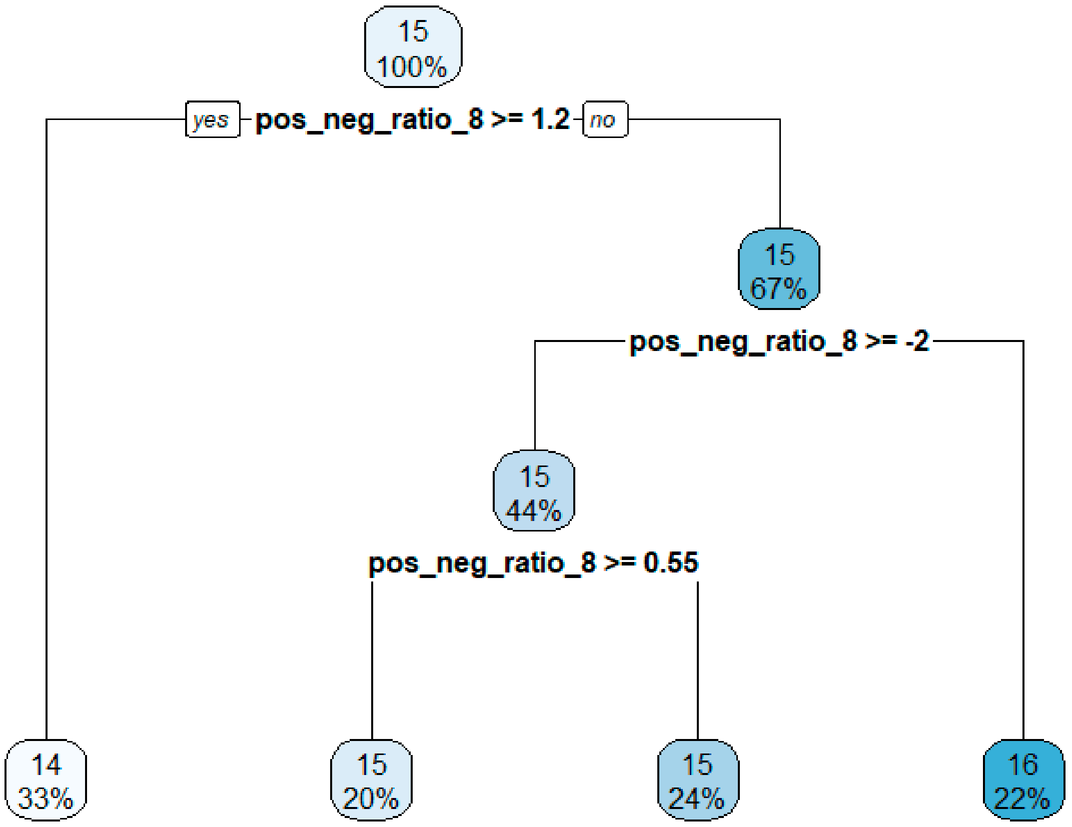

Figure S8. Regression tree trained from the training set data at pH 8.0 negative mode. In the nodes the logarithm of the response factor is found as well as the percentage of compounds belonging to that node.

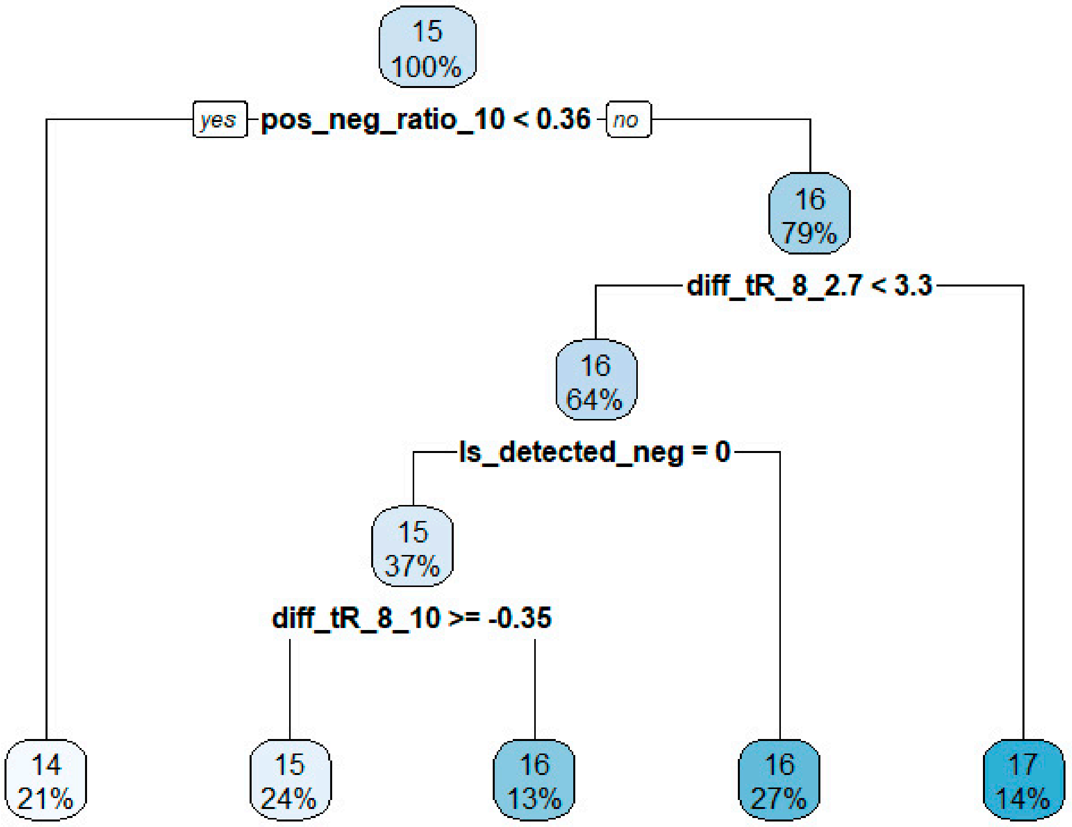

Figure S9. Regression tree trained from the training set data at pH 10.0 positive mode. In the nodes the logarithm of the response factor is found as well as the percentage of compounds belonging to that node.

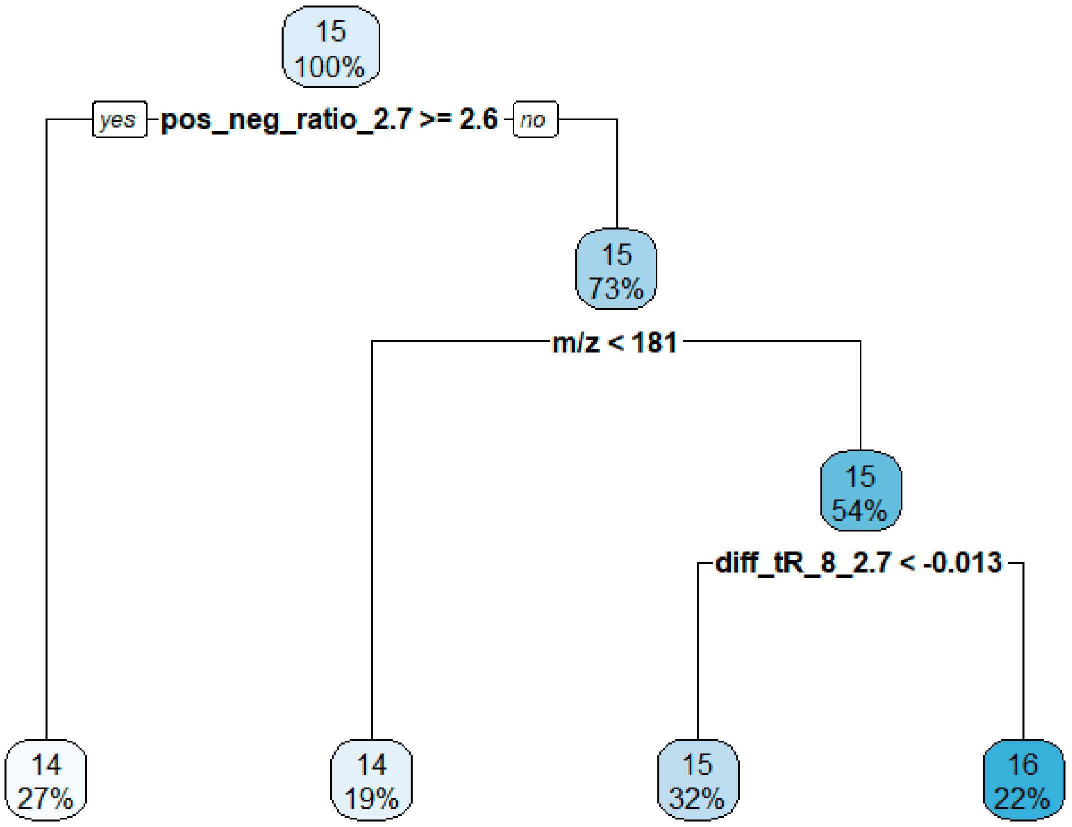

Figure S10. Regression tree trained from the training set data at pH 10.0 negative mode. In the nodes the logarithm of the response factor is found as well as the percentage of compounds belonging to that node.

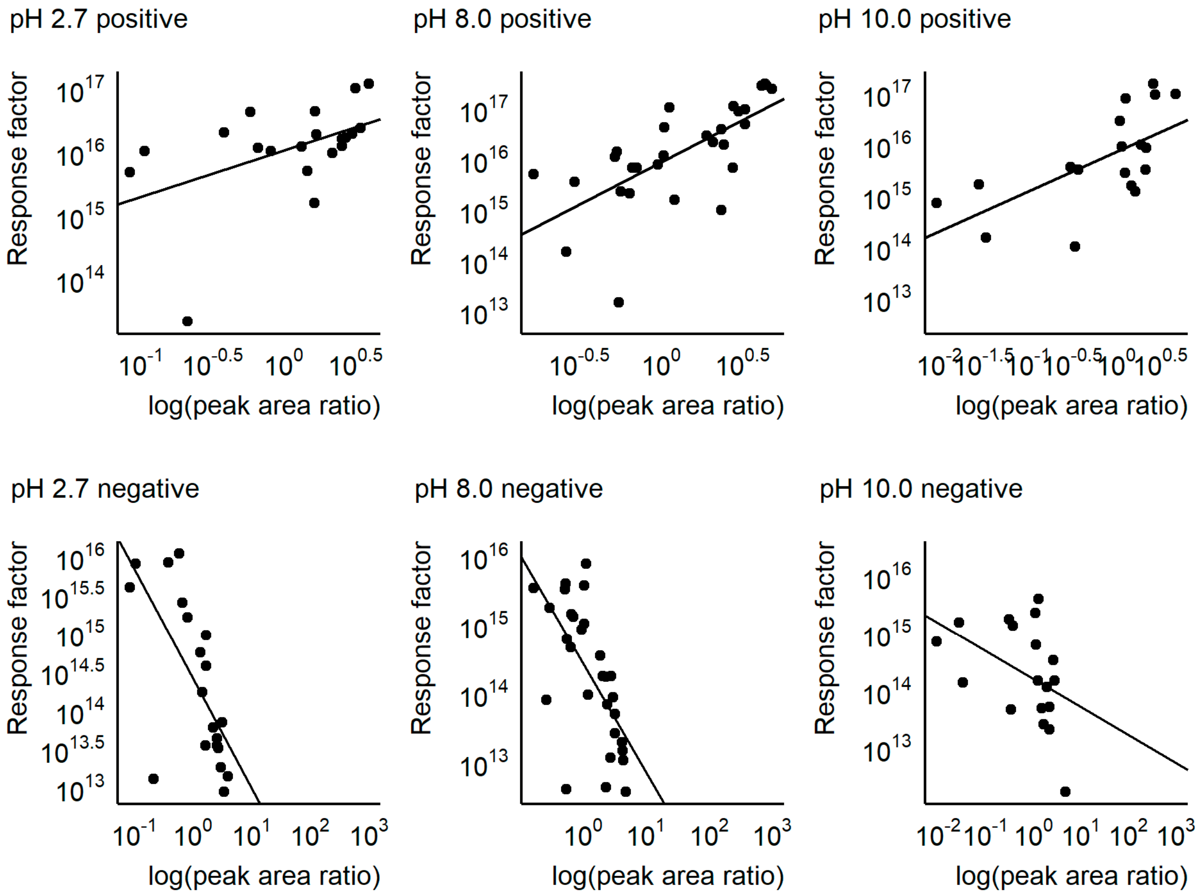

Figure S11. The response factors at each pH and mode plotted against the logarithm of the peak area ratio between positive and negative mode at the same pH. The outlier seen in pH 2.7 positive mode is Carbazole and in pH 2.7 negative mode the outlier is Chlorthalidone.

Table S1. Response factors and retention times for all pH and ionization modes as well as log*P* and pK_a for the compounds in the training set. Retention times are given in minutes. A t_R value of NA indicates that the compound was not detected.

**Compound**	**pK_a acid**	**pK_a base**	**log*P***	***RF* pH 2.7 negative**	***RF* pH 8 negative**	***RF* pH 10 negative**	***RF* pH 2.7 positive**	***RF* pH 8 positive**	***RF* pH 10 positive**	**t_R pH 2.7 negative**	**t_R pH 8 negative**	**t_R pH 10 negative**	**t_R pH 2.7 positive**	**t_R pH 8 positive**	**t_R pH 10 positive**
Acephate	10.54	NA	-0.33	$3.40 \cdot 10^{13}$	$1.00 \cdot 10^{15}$	$5.99 \cdot 10^{15}$	$1.19 \cdot 10^{16}$	$1.11 \cdot 10^{16}$	$8.73 \cdot 10^{15}$	2.92	2.47	1.52	2.89	2.45	1.51
Propylthiouracil	8.09	NA	1.204	$2.69 \cdot 10^{15}$	$3.05 \cdot 10^{15}$	$1.19 \cdot 10^{15}$	$2.08 \cdot 10^{14}$	$2.51 \cdot 10^{14}$	$1.06 \cdot 10^{13}$	4.42	3.64	1.74	4.41	3.63	1.75
Citrulline	2.27	9.23	-3.932	$1.27 * 10^{13}$	$3.93 \cdot 10^{12}$	$3.89 \cdot 10^{12}$	$2.06 \cdot 10^{13}$	$1.41 \cdot 10^{13}$	$3.20 \cdot 10^{12}$	1.66	1.71	1.48	1.66	1.71	1.51
Sulfapyridine	6.24	2.14	1.01	NA	$1.73 \cdot 10^{14}$	$2.02 \cdot 10^{13}$	$7.51 \cdot 10^{15}$	$2.09 \cdot 10^{16}$	$3.03 \cdot 10^{15}$	NA	4.10	1.91	4.28	4.10	1.90
Theophylline	7.82	-0.78	-0.77	$6.88 \cdot 10^{14}$	$1.76 \cdot 10^{15}$	$1.30 \cdot 10^{15}$	$2.19 \cdot 10^{14}$	$1.19 \cdot 10^{14}$	$3.16 \cdot 10^{13}$	1.92	3.33	1.74	1.93	3.34	1.73
Sulfamethazine	6.99	2	0.52	NA	$1.68 \cdot 10^{14}$	$4.94 \cdot 10^{13}$	$2.32 \cdot 10^{16}$	$3.75 \cdot 10^{16}$	$8.07 \cdot 10^{15}$	NA	3.98	1.91	5.08	3.97	1.90
Nicotinamide	13.39	3.63	-0.4	NA	NA	NA	$5.54 \cdot 10^{15}$	$9.83 \cdot 10^{15}$	$9.05 \cdot 10^{14}$	NA	NA	NA	1.84	1.87	1.92
Creatinine	10.45	4.96	-1.46	$5.86 \cdot 10^{13}$	$3.42 \cdot 10^{14}$	$1.09 \cdot 10^{14}$	$9.17 \cdot 10^{15}$	$2.74 \cdot 10^{16}$	$9.44 \cdot 10^{15}$	1.54	1.85	1.89	1.53	1.85	1.88
1H-Benzotriazole	9.05	0.22	1.3	$5.42 \cdot 10^{14}$	$3.30 \cdot 10^{15}$	$1.91 \cdot 10^{15}$	$1.17 \cdot 10^{16}$	$4.84 \cdot 10^{15}$	$1.53 \cdot 10^{15}$	4.84	4.27	1.87	4.83	4.26	1.87
L-lysine	NA	10.29	-3.215	NA	NA	NA	$2.53 \cdot 10^{13}$	NA	$1.95 \cdot 10^{12}$	NA	NA	NA	1.51	NA	1.88
5-Methyl-1H-benzotriazole	9.12	0.45	1.81	$3.67 \cdot 10^{14}$	$3.18 \cdot 10^{15}$	$1.28 \cdot 10^{15}$	$1.80 \cdot 10^{16}$	$1.06 \cdot 10^{16}$	$3.04 \cdot 10^{15}$	6.15	5.72	1.91	6.16	5.73	1.91
Imidazole	13.4	6.97	-0.145	NA	$1.13 \cdot 10^{13}$	NA	$3.01 \cdot 10^{15}$	$6.41 \cdot 10^{15}$	$2.82 \cdot 10^{15}$	NA	1.91	NA	1.53	1.90	1.92
Furosemide	12.68	2.3	0.56	$8.30 \cdot 10^{15}$	$1.73 \cdot 10^{15}$	$2.34 \cdot 10^{15}$	NA	NA	$2.47 \cdot 10^{13}$	8.63	4.84	4.05	NA	NA	1.91
Gabapentin	4.63	9.91	-1.27	NA	NA	NA	$9.35 \cdot 10^{15}$	$1.82 \cdot 10^{16}$	$6.68 \cdot 10^{15}$	NA	NA	NA	1.89	2.96	3.05
Metsulfuron-methyl	3.46	1.3	2.11	$8.10 \cdot 10^{15}$	$8.15 \cdot 10^{14}$	$1.67 \cdot 10^{15}$	$1.95 \cdot 10^{16}$	$7.39 \cdot 10^{15}$	$3.38 \cdot 10^{15}$	8.93	3.76	3.15	8.93	3.77	3.14
Valsartan	4.35	-0.64	5.27	$7.82 \cdot 10^{15}$	NA	$1.76 \cdot 10^{15}$	$9.98 \cdot 10^{15}$	$2.78 \cdot 10^{15}$	$1.18 \cdot 10^{15}$	12.43	NA	4.19	11.45	5.61	4.20
Pyridine	NA	5.12	0.756	NA	NA	NA	$4.58 \cdot 10^{15}$	$2.89 \cdot 10^{15}$	$1.77 \cdot 10^{15}$	NA	NA	NA	1.59	3.93	4.22
Caffeine	NA	-1.16	-0.55	NA	NA	NA	$2.43 \cdot 10^{15}$	$1.11 \cdot 10^{15}$	$1.19 \cdot 10^{14}$	NA	NA	NA	4.43	4.18	4.47
Methomyl	14	0.69	1.539	NA	NA	NA	$1.87 \cdot 10^{16}$	$8.24 \cdot 10^{15}$	$8.87 \cdot 10^{15}$	NA	NA	NA	4.46	4.14	4.55
Cotinine	NA	4.79	0.21	NA	NA	NA	$2.70 \cdot 10^{16}$	$4.66 \cdot 10^{16}$	$8.02 \cdot 10^{15}$	NA	NA	NA	1.80	4.12	4.49
Monocrotophos	15.73	-0.75	-0.46	NA	NA	NA	$4.56 \cdot 10^{16}$	$3.26 \cdot 10^{16}$	$1.76 \cdot 10^{16}$	NA	NA	NA	4.63	4.35	4.71
Aniline	NA	4.64	1.144	NA	NA	NA	$2.55 \cdot 10^{15}$	$2.56 \cdot 10^{14}$	$4.42 \cdot 10^{13}$	NA	NA	NA	1.78	4.76	4.94
Chlorthalidone	8.76	NA	1.6	$2.09 \cdot 10^{15}$	NA	$2.37 \cdot 10^{16}$	$1.43 \cdot 10^{14}$	$8.45 \cdot 10^{13}$	$3.64 \cdot 10^{13}$	6.49	NA	5.03	6.48	6.51	5.04
Phenazone	NA	0.49	1.22	NA	NA	NA	$1.03 \cdot 10^{17}$	$1.46 \cdot 10^{17}$	$9.99 \cdot 10^{16}$	NA	NA	NA	5.46	5.10	5.63
Quinoxaline	NA	1.86	1.3	NA	NA	NA	$1.14 \cdot 10^{15}$	$3.44 \cdot 10^{14}$	$1.24 \cdot 10^{14}$	NA	NA	NA	5.93	5.75	6.00
fluconazole	12.68	2.3	0.561	$1.55 \cdot 10^{15}$	$3.82 \cdot 10^{15}$	$1.49 \cdot 10^{15}$	$9.98 \cdot 10^{15}$	$1.32 \cdot 10^{16}$	$1.58 \cdot 10^{15}$	5.83	5.78	5.95	5.84	5.79	5.94

Imidacloprid	9.39	5.28	1.101	$1.68 \cdot 10^{14}$	$1.72 \cdot 10^{15}$	$1.04 \cdot 10^{15}$	$4.79 \cdot 10^{15}$	$3.40 \cdot 10^{15}$	$7.64 \cdot 10^{14}$	6.05	5.82	6.17	6.06	5.81	6.18
Chloridazon	NA	-1.77	1.11	$9.18 \cdot 10^{14}$	$3.59 \cdot 10^{15}$	$2.18 \cdot 10^{15}$	$4.17 \cdot 10^{16}$	$4.07 \cdot 10^{16}$	$2.76 \cdot 10^{16}$	6.09	5.94	6.21	6.10	5.95	6.22
Benzylamine	NA	9.3	1.099	NA	NA	NA	$2.59 \cdot 10^{15}$	$3.29 \cdot 10^{15}$	$2.14 \cdot 10^{15}$	NA	NA	NA	1.87	4.17	6.35
Trimethoprim	NA	7.16	1.28	$8.61 \cdot 10^{12}$	NA	NA	$2.28 \cdot 10^{16}$	$5.72 \cdot 10^{16}$	$4.94 \cdot 10^{16}$	10.37	NA	NA	3.54	6.41	6.71
Triethyl phosphate	NA	NA	1.18	NA	NA	NA	$5.17 \cdot 10^{16}$	$2.73 \cdot 10^{16}$	$2.56 \cdot 10^{16}$	NA	NA	NA	6.82	6.51	6.96
Irbesartan	5.85	4.12	5.39	$1.06 \cdot 10^{16}$	$7.44 \cdot 10^{15}$	$3.81 \cdot 10^{15}$	$4.09 \cdot 10^{16}$	$9.95 \cdot 10^{16}$	$7.41 \cdot 10^{16}$	8.96	8.06	7.02	8.95	8.04	7.01
Butylamine	NA	10.21	0.698	NA	NA	NA	$1.48 \cdot 10^{15}$	$2.17 \cdot 10^{15}$	$1.10 \cdot 10^{15}$	NA	NA	NA	1.64	3.10	7.32
Glimepiride	4.32	NA	3.12	$3.84 \cdot 10^{15}$	$1.01 \cdot 10^{15}$	$6.72 \cdot 10^{14}$	$4.66 \cdot 10^{15}$	$9.50 \cdot 10^{14}$	$6.87 \cdot 10^{14}$	12.72	8.86	7.48	12.72	8.84	7.48
Benzothiazole	NA	2.28	2.11	NA	NA	NA	$2.25 \cdot 10^{15}$	$6.35 \cdot 10^{14}$	$2.86 \cdot 10^{14}$	NA	NA	NA	7.81	7.72	7.99
Monuron	13.44	NA	1.93	$3.19 \cdot 10^{13}$	$1.27 \cdot 10^{15}$	$1.41 \cdot 10^{14}$	$1.62 \cdot 10^{16}$	$6.54 \cdot 10^{15}$	$2.64 \cdot 10^{15}$	8.11	8.06	8.20	8.12	8.05	8.21
Phenylethylamine	NA	NA	4.15	NA	NA	NA	$8.05 \cdot 10^{15}$	$1.33 \cdot 10^{16}$	$9.64 \cdot 10^{15}$	NA	NA	NA	1.89	5.18	8.27
Simazine	14.75	4.23	1.78	NA	$2.54 \cdot 10^{13}$	NA	$2.13 \cdot 10^{16}$	$4.70 \cdot 10^{16}$	$2.98 \cdot 10^{16}$	NA	8.02	NA	8.02	8.01	8.15
Cyanazine	14.44	0.57	1.96	$1.80 \cdot 10^{13}$	$1.36 \cdot 10^{15}$	$1.41 \cdot 10^{14}$	$1.86 \cdot 10^{16}$	$6.39 \cdot 10^{15}$	$8.80 \cdot 10^{16}$	8.29	8.25	8.43	8.28	8.26	8.42
Iminostilbene	NA	0.34	3.78	NA	$4.22 \cdot 10^{12}$	NA	$1.21 \cdot 10^{16}$	$9.53 \cdot 10^{14}$	$3.96 \cdot 10^{15}$	NA	12.33	NA	12.40	12.34	8.85
Atraton	14.44	6	0.62	$1.38 \cdot 10^{13}$	$3.61 \cdot 10^{12}$	NA	$1.15 \cdot 10^{17}$	$2.30 \cdot 10^{17}$	$1.61 \cdot 10^{17}$	1.90	8.68	NA	5.18	8.67	8.89
1-Nitropyrene	NA	NA	4.22	NA	$7.81 \cdot 10^{13}$	$1.32 \cdot 10^{14}$	$2.32 \cdot 10^{13}$	$1.43 \cdot 10^{14}$	$1.43 \cdot 10^{14}$	NA	9.18	9.01	9.16	9.17	9.02
Carbamazepine	NA	-1.58	2.62	NA	NA	NA	$5.22 \cdot 10^{16}$	$5.44 \cdot 10^{16}$	$1.95 \cdot 10^{16}$	NA	NA	NA	8.73	8.53	8.85
Corticosterone	13.86	-0.26	2.02	NA	$1.49 \cdot 10^{15}$	$7.43 \cdot 10^{13}$	$1.10 \cdot 10^{15}$	$4.69 \cdot 10^{14}$	$4.86 \cdot 10^{13}$	9.36	9.39	9.44	9.36	9.39	9.45
Atrazine	4.2	14.48	2.2	NA	$4.88 \cdot 10^{13}$	NA	$4.63 \cdot 10^{16}$	$9.11 \cdot 10^{16}$	$5.28 \cdot 10^{16}$	NA	9.50	NA	9.45	9.49	9.61
O-Desmethylvenlafaxine	10.11	8.87	2.29	NA	$8.41 \cdot 10^{13}$	NA	$3.02 \cdot 10^{16}$	$8.29 \cdot 10^{16}$	$8.32 \cdot 10^{15}$	NA	7.44	NA	3.93	7.43	9.84
Diuron	13.18	NA	2.53	$2.42 \cdot 10^{15}$	$8.66 \cdot 10^{15}$	$4.99 \cdot 10^{15}$	$1.10 \cdot 10^{16}$	$2.52 \cdot 10^{15}$	$7.31 \cdot 10^{14}$	9.92	9.97	10.01	9.92	9.97	10.00
Hydrocortisonacetate	12.61	NA	1.71	$3.43 \cdot 10^{13}$	$9.12 \cdot 10^{14}$	$4.44 \cdot 10^{13}$	$1.51 \cdot 10^{15}$	$4.72 \cdot 10^{14}$	$9.77 \cdot 10^{13}$	9.98	10.04	10.11	10.00	10.03	10.10
9.10-Phenanthrenequinone	NA	NA	2.92	NA	NA	NA	$3.28 \cdot 10^{15}$	$9.60 \cdot 10^{14}$	$7.75 \cdot 10^{14}$	NA	NA	NA	10.06	9.85	10.19
Metazachlor	NA	2.34	2.98	NA	NA	NA	$5.60 \cdot 10^{16}$	$3.95 \cdot 10^{16}$	$2.26 \cdot 10^{16}$	NA	NA	NA	10.30	10.16	10.45
Ametryn	14.63	6.74	2.6	NA	$1.43 \cdot 10^{13}$	NA	$1.45 \cdot 10^{17}$	$2.97 \cdot 10^{17}$	$1.81 \cdot 10^{17}$	NA	10.68	NA	7.34	10.68	10.83
Norethindrone	NA	-1.66	3.22	NA	NA	NA	$2.22 \cdot 10^{15}$	$7.22 \cdot 10^{14}$	$8.87 \cdot 10^{13}$	NA	NA	NA	10.81	10.76	10.91
Estrone	10.33	NA	4.31	NA	$3.06 \cdot 10^{14}$	NA	NA	$5.02 \cdot 10^{13}$	NA	NA	11.09	NA	NA	11.07	11.10
2-(Methylthio)benzothiazole	NA	1.13	3.42	NA	NA	NA	$4.41 \cdot 10^{15}$	$1.45 \cdot 10^{15}$	$9.33 \cdot 10^{14}$	NA	NA	NA	11.24	11.17	11.38
Sebuthylazine	14.4	4.2	2.72	NA	$6.65 \cdot 10^{13}$	NA	$2.99 \cdot 10^{16}$	$1.83 \cdot 10^{16}$	$6.58 \cdot 10^{16}$	11.54	11.46	NA	11.56	11.46	10.81

Prometryn	13.9	NA	3.85	NA	$1.03 \cdot 10^{13}$	NA	$1.46 \cdot 10^{17}$	$2.77 \cdot 10^{17}$	$1.75 \cdot 10^{17}$	NA	11.88	NA	8.58	11.88	12.04
Ethoprop	NA	NA	3.22	$4.25 \cdot 10^{13}$	NA	NA	$1.54 \cdot 10^{16}$	$4.65 \cdot 10^{15}$	$3.45 \cdot 10^{15}$	8.18	NA	NA	11.97	11.88	12.12
Climbazole	NA	6.49	4.33	$6.75 \cdot 10^{13}$	NA	$3.23 \cdot 10^{14}$	$9.67 \cdot 10^{16}$	$1.53 \cdot 10^{17}$	$1.47 \cdot 10^{17}$	7.57	NA	12.24	7.58	12.13	12.25
Irgarol	14.13	6.68	2.98	NA	$1.90 \cdot 10^{13}$	NA	$1.45 \cdot 10^{17}$	$2.73 \cdot 10^{17}$	$1.84 \cdot 10^{17}$	NA	12.19	NA	9.32	12.19	12.35
Metolachlor	NA	NA	3.45	NA	NA	NA	$2.64 \cdot 10^{16}$	$1.46 \cdot 10^{16}$	$7.95 \cdot 10^{15}$	NA	NA	NA	12.41	12.33	12.58
Alachlor	NA	NA	3.59	NA	NA	NA	$3.36 \cdot 10^{15}$	$1.22 \cdot 10^{15}$	$1.05 \cdot 10^{15}$	NA	NA	NA	12.44	12.38	12.60
Efavirenz	12.52	-1.49	4.46	$5.01 \cdot 10^{15}$	$1.06 \cdot 10^{16}$	$9.61 \cdot 10^{15}$	$5.31 \cdot 10^{13}$	$2.97 \cdot 10^{13}$	$1.16 \cdot 10^{13}$	12.78	12.95	12.72	12.78	12.95	12.73
Ketoconazole	NA	6.42	4.19	NA	NA	NA	$2.98 \cdot 10^{16}$	$3.91 \cdot 10^{16}$	$5.06 \cdot 10^{16}$	NA	NA	NA	7.40	12.38	12.66
Imazalil	NA	6.48	3.76	NA	NA	NA	$1.32 \cdot 10^{17}$	$2.25 \cdot 10^{17}$	$2.05 \cdot 10^{17}$	NA	NA	NA	6.90	12.66	12.85
Haloperidol	13.96	8.05	3.66	NA	$1.71 \cdot 10^{14}$	$1.66 \cdot 10^{12}$	$7.02 \cdot 10^{16}$	$1.06 \cdot 10^{17}$	$9.22 \cdot 10^{16}$	NA	12.55	12.88	6.58	12.54	12.89
Ethyl_azinphos	NA	NA	3.958	NA	NA	NA	$2.02 \cdot 10^{15}$	$5.47 \cdot 10^{14}$	$2.25 \cdot 10^{14}$	NA	NA	NA	12.77	12.69	12.91
Progesterone	14.46	6.71	3.03	NA	NA	NA	$2.44 \cdot 10^{15}$	$6.38 \cdot 10^{14}$	$1.07 \cdot 10^{14}$	NA	NA	NA	13.14	12.91	13.26
Sudan III	11.34	0.22	5.76	NA	$1.90 \cdot 10^{13}$	NA	NA	NA	NA	NA	13.26	NA	NA	NA	13.67
Reserpine	NA	7.02	3.53	NA	$5.97 \cdot 10^{14}$	$2.50 \cdot 10^{13}$	$3.56 \cdot 10^{15}$	$2.20 \cdot 10^{15}$	$1.14 \cdot 10^{15}$	7.88	13.36	13.78	7.87	13.36	13.77
Tri-isobutyl-phosphate	NA	NA	3.85	NA	NA	NA	$5.38 \cdot 10^{16}$	$2.11 \cdot 10^{16}$	$2.66 \cdot 10^{16}$	NA	NA	NA	13.60	13.50	13.77
Triphenylphosphate	NA	NA	5.09	NA	NA	NA	$7.12 \cdot 10^{15}$	$3.21 \cdot 10^{15}$	$3.32 \cdot 10^{15}$	NA	NA	NA	13.69	13.49	13.84
Clotrimazole	NA	6.26	5.839	NA	NA	NA	$2.32 \cdot 10^{16}$	$3.90 \cdot 10^{16}$	$3.88 \cdot 10^{16}$	NA	NA	NA	8.06	13.79	14.19
Simvastatin	11.84	-0.1	5.06	NA	$9.23 \cdot 10^{13}$	NA	$6.02 \cdot 10^{15}$	$1.51 \cdot 10^{15}$	$2.86 \cdot 10^{15}$	NA	14.52	NA	14.67	14.52	14.81
Sudan I	11.84	-0.1	5.065	NA	$4.49 \cdot 10^{14}$	$4.75 \cdot 10^{13}$	$1.73 \cdot 10^{16}$	$1.99 \cdot 10^{15}$	$1.50 \cdot 10^{15}$	NA	14.96	15.38	15.25	14.95	15.37
Chlorpyrifos	NA	NA	4.78	NA	NA	NA	$3.43 \cdot 10^{13}$	NA	NA	NA	NA	NA	15.52	NA	15.65
Trifluralin	NA	NA	4.6	NA	NA	NA	NA	NA	NA	NA	NA	NA	NA	NA	17.95
Cyclamic acid	-0.83	NA	0.607	$9.03 \cdot 10^{15}$	$2.90 \cdot 10^{15}$	$2.75 \cdot 10^{15}$	NA	NA	NA	5.96	1.88	1.77	NA	NA	NA
4-Amino-6-chloro-1.3-benzenedisulfonamide	9.19	-0.83	-1.04	$2.01 \cdot 10^{15}$	$2.17 \cdot 10^{15}$	$3.05 \cdot 10^{15}$	NA	NA	NA	3.49	3.41	1.80	NA	NA	NA
Gemfibrozil	4.42	NA	4.39	$1.98 \cdot 10^{14}$	$3.42 \cdot 10^{15}$	$5.55 \cdot 10^{14}$	NA	$5.69 \cdot 10^{12}$	NA	13.27	8.37	6.54	NA	8.38	NA
Chloramphenicol	8.69	NA	0.879	$1.12 \cdot 10^{16}$	$2.99 \cdot 10^{16}$	$2.18 \cdot 10^{16}$	NA	NA	NA	6.91	7.11	6.95	NA	NA	NA
1-Naphthol	9.6	NA	2.659	$7.62 \cdot 10^{12}$	$2.15 \cdot 10^{15}$	$1.81 \cdot 10^{12}$	NA	NA	NA	9.59	9.78	8.84	NA	NA	NA
Linoleic acid	4.99	NA	6.422	$3.80 \cdot 10^{13}$	$3.08 \cdot 10^{15}$	$1.42 \cdot 10^{14}$	NA	NA	NA	17.46	12.56	10.28	NA	NA	NA
Propanil	8.09	NA	1.02	$2.51 \cdot 10^{16}$	NA	$2.10 \cdot 10^{16}$	NA	NA	NA	10.80	NA	10.88	NA	NA	NA
Bicalutamide	11.78	NA	2.71	$4.26 \cdot 10^{16}$	$8.41 \cdot 10^{16}$	$5.14 \cdot 10^{16}$	NA	NA	NA	11.16	11.33	11.25	NA	NA	NA

1-hydroxypyrene	9.5	NA	3.98	$4.19 \cdot 10^{15}$	$7.20 \cdot 10^{15}$	$-6.01 \cdot 10^{13}$	$1.99 \cdot 10^{13}$	$2.17 \cdot 10^{13}$	NA	12.54	12.52	11.70	12.55	12.52	NA
Carbazole	NA	14.97	3.09	NA	$1.40 \cdot 10^{15}$	$1.62 \cdot 10^{13}$	$6.00 \cdot 10^{12}$	$5.45 \cdot 10^{13}$	NA	NA	11.79	11.84	13.62	13.52	NA
Fipronil	NA	0.18	4.49	$7.89 \cdot 10^{16}$	$1.76 \cdot 10^{17}$	$8.58 \cdot 10^{16}$	NA	NA	NA	13.19	13.40	13.27	NA	NA	NA
Tyramine	10.41	9.66	0.68	NA	NA	NA	$2.26 \cdot 10^{14}$	$-1.11 \cdot 10^{14}$	NA	NA	3.21	NA	1.71	3.22	NA
2.2-Dipyridyl	NA	3.3	1.957	NA	NA	NA	$3.41 \cdot 10^{15}$	NA	NA	NA	NA	NA	3.09	NA	NA
4-Chloroaniline	NA	3.49	1.75	NA	$7.33 \cdot 10^{12}$	NA	$6.77 \cdot 10^{14}$	NA	NA	NA	8.02	NA	3.73	NA	NA
Parathion	NA	NA	3.32	NA	NA	NA	NA	NA	NA	NA	NA	NA	13.45	NA	NA
Dicamba	2.54	NA	2.681	$2.32 \cdot 10^{15}$	$1.69 \cdot 10^{14}$	NA	NA	NA	NA	8.84	1.88	NA	NA	NA	NA
17beta-estradiol	10.33	-0.88	3.74	NA	$1.35 \cdot 10^{14}$	NA	NA	NA	NA	NA	10.68	NA	NA	NA	NA
Abietic acid	4.59	NA	4.95	NA	$1.85 \cdot 10^{14}$	NA	NA	NA	NA	NA	11.99	NA	NA	NA	NA

Table S2. Response factors and retention times for all pH and ionization modes as well as log*P* and pK_a for the compounds in the testset. Retention times are given in minutes. A t_R value of NA indicates that the compound was not detected.

**Compound name**	**pK_a acid**	**pK_a base**	**log*P***	***RF* pH 2.7 negative**	***RF* pH 8 negative**	***RF* pH 10 negative**	***RF* pH 2.7 positive**	***RF* pH 8 positive**	***RF* pH 10 positive**	**t_R pH 2.7 negative**	**t_R pH 8 negative**	**t_R pH 10 negative**	**t_R pH 2.7 positive**	**t_R pH 8 positive**	**t_R pH 10 positive**
(S)-3-Anilino-5-methyl-5-phenylimidazolidine-2,4-dione	10.87		3.021	NA	$1.2 \cdot 10^{15}$	$2.8 \cdot 10^{14}$	$4.9 \cdot 10^{14}$	$4.0 \cdot 10^{14}$	$1.3 \cdot 10^{14}$	NA	9.11	9.22	9.11	9.10	9.19
2,6-Diaminopyridin		8.72	0.287	NA	NA	NA	$3.7 \cdot 10^{16}$	$8.6 \cdot 10^{16}$	$1.4 \cdot 10^{15}$	NA	NA	NA	1.66	2.85	2.94
5,5-Diphenylhydantoin	8.49		2.148	$6.0 \cdot 10^{13}$	$1.1 \cdot 10^{15}$	$1.1 \cdot 10^{14}$	$1.1 \cdot 10^{13}$	NA	NA	8.65	8.58	4.69	8.65	8.59	NA
Acridine		6.15	3.506	NA	NA	NA	$4.4 \cdot 10^{16}$	$8.4 \cdot 10^{16}$	$5.2 \cdot 10^{16}$	NA	NA	NA	4.18	10.44	10.82
Ampicillin	3.24	7.23	-2.005	$4.0 \cdot 10^{15}$	$1.4 \cdot 10^{15}$	$1.5 \cdot 10^{15}$	$8.9 \cdot 10^{15}$	$1.5 \cdot 10^{15}$	$1.6 \cdot 10^{15}$	3.67	4.00	3.31	3.68	4.01	3.30
anziphos-ethyl			3.958	NA	NA	NA	$1.9 \cdot 10^{15}$	$8.0 \cdot 10^{14}$	$3.3 \cdot 10^{14}$	NA	NA	NA	12.76	12.69	12.93
benzylamine		9.3	1.099	NA	NA	NA	$4.8 \cdot 10^{15}$	$6.0 \cdot 10^{15}$	$3.5 \cdot 10^{15}$	NA	NA	NA	1.86	4.17	6.36
cyanazine	14.44	0.57	1.962	NA	$9.8 \cdot 10^{14}$	$1.2 \cdot 10^{14}$	$1.5 \cdot 10^{16}$	$3.9 \cdot 10^{15}$	$1.3 \cdot 10^{15}$	8.21	8.26	8.42	8.29	8.24	8.41
Danofloxacin	5.49	7.31	0.063	NA	NA	NA	$2.0 \cdot 10^{16}$	$1.3 \cdot 10^{16}$	$6.6 \cdot 10^{15}$	NA	NA	NA	4.41	8.19	4.23
dazomet		4.06	1.283	NA	NA	NA	$4.3 \cdot 10^{15}$	$5.2 \cdot 10^{14}$	$3.5 \cdot 10^{14}$	NA	NA	NA	4.41	4.18	4.52
diazinon		4.19	4.192	NA	NA	NA	$1.2 \cdot 10^{17}$	$7.9 \cdot 10^{16}$	$1.4 \cdot 10^{17}$	NA	NA	NA	13.65	13.61	13.88
Diclofenac	4.00		4.259	$2.0 \cdot 10^{16}$	$6.1 \cdot 10^{15}$	$4.5 \cdot 10^{15}$	$7.6 \cdot 10^{14}$	$3.0 \cdot 10^{14}$	$2.6 \cdot 10^{13}$	12.17	7.70	6.31	12.18	7.56	5.84
dimethyl phtalate			1.98	NA	NA	NA	$7.3 \cdot 10^{14}$	$2.5 \cdot 10^{14}$	$3.1 \cdot 10^{14}$	NA	NA	NA	8.37	8.17	8.54
diphenyl phthalate			5.296	NA	NA	NA	$6.5 \cdot 10^{14}$	$4.4 \cdot 10^{14}$	$4.2 \cdot 10^{14}$	NA	NA	NA	14.04	13.85	14.22
febantel	9.48		3.166	NA	NA	NA	$1.4 \cdot 10^{17}$	$2.1 \cdot 10^{17}$	$1.3 \cdot 10^{17}$	NA	NA	NA	12.48	12.40	12.58
imidazole	13.4	6.97	-0.145	NA	$1.8 \cdot 10^{13}$	NA	$5.5 \cdot 10^{15}$	$1.3 \cdot 10^{16}$	$3.7 \cdot 10^{15}$	NA	1.90	NA	1.52	1.89	1.91
ketoprofen	3.88		3.613	$4.0 \cdot 10^{15}$	$1.1 \cdot 10^{15}$	$1.0 \cdot 10^{15}$	$5.9 \cdot 10^{14}$	$1.7 \cdot 10^{15}$	$8.5 \cdot 10^{14}$	10.34	5.88	4.73	10.33	5.53	4.74
matalaxyl	15.8		2.12	NA	NA	NA	$1.3 \cdot 10^{17}$	$7.5 \cdot 10^{16}$	$5.3 \cdot 10^{16}$	NA	NA	NA	9.74	9.55	9.94
methidathion			2.293	NA	NA	NA	$4.0 \cdot 10^{14}$	$1.7 \cdot 10^{14}$	$1.1 \cdot 10^{14}$	NA	NA	NA	11.05	11.00	11.19
methomyl	14.0	0.69	0.72	NA	NA	NA	$2.0 \cdot 10^{16}$	$8.6 \cdot 10^{15}$	$1.0 \cdot 10^{16}$	NA	NA	NA	4.47	4.15	4.62
Mianserin	6.92		3.831	NA	NA	NA	$9.9 \cdot 10^{16}$	$1.4 \cdot 10^{17}$	$2.1 \cdot 10^{17}$	NA	NA	NA	6.21	13.35	13.77
miconazole nitrate	6.48		5.956	NA	NA	NA	$9.2 \cdot 10^{16}$	$1.7 \cdot 10^{17}$	$2.0 \cdot 10^{17}$	NA	NA	NA	9.29	15.39	15.82
Naproxen	4.19		2.986	$2.8 \cdot 10^{13}$	$2.6 \cdot 10^{14}$	$6.1 \cdot 10^{13}$	$7.2 \cdot 10^{12}$	NA	NA	10.34	5.47	4.40	10.35	NA	NA
Ofloxacin	5.35	6.72	0.09	NA	$1.6 \cdot 10^{14}$	NA	$1.7 \cdot 10^{16}$	$2.6 \cdot 10^{16}$	$1.9 \cdot 10^{16}$	NA	5.74	NA	4.07	5.95	3.75
propanil	13.9		3.12	$2.7 \cdot 10^{16}$	$1.3 \cdot 10^{16}$	$2.1 \cdot 10^{16}$	$6.5 \cdot 10^{14}$	$9.2 \cdot 10^{13}$	$4.9 \cdot 10^{13}$	10.78	10.92	10.90	10.80	10.92	10.89
Quinine	13.89	9.05	2.513	NA	$1.0 \cdot 10^{14}$	NA	$4.5 \cdot 10^{16}$	$5.5 \cdot 10^{16}$	$6.1 \cdot 10^{16}$	NA	10.67	NA	3.76	10.68	11.34

tetramethylthioram disulfide			2.733	NA	NA	NA	$1.9 \cdot 10^{16}$	$1.5 \cdot 10^{16}$	$9.4 \cdot 10^{15}$	NA	NA	NA	13.50	13.27	13.65
thiabendazole	10.28	4.08	2.329	NA	NA	$7.0 \cdot 10^{13}$	$4.2 \cdot 10^{16}$	$2.9 \cdot 10^{17}$	$1.1 \cdot 10^{17}$	NA	NA	7.41	3.66	7.28	7.42

Table S3. Retention times for all pH and ionization modes as well as log*P* and pK_a for the compounds in the validation set. Retention times are given in minutes. A t_R value of NA indicates that the compound was not detected.

**Compound name**	**pK_a acid**	**pK_a base**	**log*P***	**t_R pH 2.7 negative**	**t_R pH 8 negative**	**t_R pH 10 negative**	**t_R pH 2.7 positive**	**t_R pH 8 positive**	**t_R pH 10 positive**
Histamine	14.46	9.58	-0.701	NA	NA	1.20	1.48	2.63	NA
Guanylurea	13.62	9.79	-2.034	NA	1.99	NA	1.53	1.98	1.86
Metformin		12.3	-0.918	NA	NA	NA	1.52	2.46	5.59
Amitrole	11.11	3.55	-0.559	NA	1.87	NA	1.54	1.87	1.71
Atrazine-desethyl-desisopropyl-2-hydroxy	5.74	1.55	-2.505	1.54	1.80	1.52	1.53	1.80	1.51
Butylamine		10.21	0.698	NA	NA	NA	1.62	NA	NA
Atrazine-desisopropyl-2-hydroxy	5.82	3.25	0.589	1.64	2.40	1.97	1.64	2.40	1.96
Atrazine-desethyl-2-hydroxy	5.74	1.55	-2.505	1.81	3.06	2.61	1.80	3.07	2.61
Adenosine	12.45	3.94	-2.091	NA	2.91	NA	1.96	2.91	3.05
Methamidophos	15.57		-0.318	NA	NA	NA	2.52	2.41	2.53
Atrazine-desethyl-desisopropyl	15.26	4.58	0.18	NA	NA	NA	2.73	2.70	2.75
2-Aminobenzothiazole		4.48	1.969	NA	NA	NA	3.14	6.81	6.75
Vancomycin	3.38	9.8	-4.386	NA	NA	NA	3.09	NA	NA
Omethoate	15.46		-0.548	NA	NA	NA	3.28	3.06	3.29
Atrazine-2-hydroxy	5.8	3.56	-0.436	3.43	5.92	5.77	3.43	5.92	5.78
Chlorothiazide	9.19		-0.442	3.82	2.65	1.46	3.81	NA	1.49
Benzotriazole-5-carboxylic acid	3.6	-0.33	0.959	4.21	1.61	1.37	4.21	1.61	1.39
Caffeine		-1.16	-0.546	NA	NA	NA	4.42	4.19	4.42
Caffeine-13C3		-1.16	-0.546	NA	NA	NA	4.42	4.20	4.44
Atrazine-desisopropyl	14.94	4.41	1.123	NA	NA	NA	7.11	7.14	7.16
Benzotriazole	9.04	0.22	1.301	4.84	4.28	1.88	4.84	4.28	1.87
Trichlorfon	10.12		1.138	NA	NA	NA	5.36	NA	NA
Atrazine-desethyl	14.58	4.38	1.539	NA	NA	NA	6.05	6.09	6.09
5-Methyl-1H-benzotriazole	9.12	0.45	1.815	6.23	5.93	NA	6.24	5.92	2.85
Haloperidol	13.96	8.05	3.661	NA	NA	NA	6.54	12.58	12.79

10.11-Dihydro-10-hydroxycarbamazepine	14.1		1.732	NA	NA	NA	6.68	6.57	6.72
5-Chlorobenzotriazole	9.04	0.1	1.905	6.96	5.52	2.89	6.96	NA	NA
Butocarboxim	14.33	1.33	1.331	NA	NA	NA	7.04	NA	7.08
Imazalil-d5		6.48	3.755	NA	NA	NA	6.84	12.64	12.70
Imazalil		6.48	3.755	NA	NA	NA	6.86	12.69	12.74
Tylosin	12.45	8.43	2.316	NA	11.52	NA	7.24	11.51	11.54
Carbamazepine-10.11-epoxide	15.96		1.974	NA	NA	NA	7.42	7.15	7.46
Ketoconazole		6.42	4.192	NA	NA	NA	7.37	12.41	12.55
Climbazole		6.49	4.335	NA	12.13	NA	7.54	12.14	12.14
Benzothiazole		2.28	2.113	NA	NA	NA	7.79	7.69	7.85
Clarithromycin	12.46	9	3.24	NA	13.99	NA	7.68	13.96	14.94
Simazine	14.75	4.23	1.781	NA	NA	NA	8.01	8.05	8.08
Clotrimazole		6.26	5.839	NA	1.49	NA	8.03	13.82	14.08
Reserpine	7.02		3.531	NA	13.38	NA	7.83	13.38	13.66
Dichlorvos			1.373	NA	NA	NA	8.22	NA	8.26
Carbamazepine	15.96		2.766	NA	NA	NA	8.72	8.55	8.76
2-Methylbenzothaizole		2.97	2.237	NA	NA	NA	8.96	8.92	9.04
Irgarol	14.13	6.68	2.985	NA	NA	NA	9.32	12.20	12.25
Atrazine-d5	14.48	4.2	2.198	NA	NA	NA	9.40	9.46	9.46
Atrazine	14.48	4.2	2.198	NA	9.52	NA	9.45	9.51	9.51
Phenazine		2.7	3.06	NA	NA	NA	9.48	9.33	9.51
Spinosad		9.17	5.843	NA	NA	NA	9.62	17.11	17.91
Metazachlor		2.34	2.979	NA	NA	NA	10.29	10.18	10.33
Emamectin	12.55	9.34	6.174	NA	16.81	NA	10.54	16.79	17.16
2-(Methylthio)benzothiazole		1.13	3.426	NA	NA	NA	11.23	NA	11.27
TCMTB		1.02	3.689	NA	NA	NA	11.70	NA	NA

Metolachlor-OXA	3.21		2.876	12.12	5.72	4.67	12.09	NA	4.52
Rifaximin	6.69	5.88	4.589	NA	NA	NA	12.14	NA	NA
Metolachlor			3.453	NA	NA	NA	12.40	12.35	12.46
Efavirenz	12.52	-1.49	4.457	12.76	12.96	12.61	12.76	NA	NA
Progesterone			4.149	NA	NA	NA	13.13	NA	NA
Metolachlor-ESA	-0.68		2.114	14.29	6.23	5.12	14.32	NA	NA
Simvastatin	14.91		4.458	NA	NA	NA	14.66	NA	14.69
Sudan I	11.84	-0.1	5.065	NA	NA	NA	15.25	NA	15.26
Chlorpyrifos			4.784	NA	NA	NA	15.51	NA	NA
Octocrylene			6.783	NA	NA	NA	17.13	NA	NA
Sucralose	11.91		-0.465	4.73	4.76	4.70	NA	NA	NA
2-Hydroxybenzothiazole	11.27	-1.29	2.494	7.07	7.06	NA	NA	NA	NA
Nigericin	4.07		6.107	19.87	17.79	19.13	NA	NA	NA
Aspartame	3.53	8.53	-2.218	NA	4.19	NA	NA	4.20	3.05

Table S4. Hyper parameters for the LC/MS models trained with manually integrated data.

Parameter	Positive mode pH 2.7	Negative mode pH 2.7	Positive mode pH 8	Negative mode pH 8	Positive mode pH 10	Negative mode pH 10
mtry	7	12	7	7	7	12
coefReg	0.505	1	0.505	0.505	1	0.505
coefImp	0.5	0.5	0.5	0.5	0.5	0.5

Table S5. All evaluation metrics. Mean. median and maximum error factor as well as the percentage of datapoints with a prediction error lower than a factor of 10 for all tested models: LC/MS features, Equal RF, closest eluting standard and 2D structural features[11] at each pH and ionization mode for the training set.Green colour indicates better performance.

Dataset	pH	model	Mode	Mean	Median	Max	% less than 10
Training	2.7	LC/MS features	Positive	2.2	1.6	80.4	99.00%
Training	2.7	LC/MS features	Negative	2.6	1.7	20.3	97.80%
Training	8.0	LC/MS features	Positive	3.71	1.7	420	98.70%
Training	8.0	LC/MS features	Negative	2.7	1.5	96.4	97.40%
Training	10.0	LC/MS features	Positive	3.8	1.9	216	97.40%
Training	10.0	LC/MS features	Negative	4.1	1.8	115.7	95.10%
Training	2.7	2D structural features	Positive	22.5	3.1	1892.3	83.80%
Training	2.7	2D structural features	Negative	13.6	5.3	139.2	62.90%
Training	8.0	2D structural features	Positive	20.6	5.8	528.6	70.90%
Training	8.0	2D structural features	Negative	23.2	5.1	545.5	68.30%
Training	10.0	2D structural features	Positive	27.9	4.3	948.8	73.00%
Training	10.0	2D structural features	Negative	27.7	4.5	967.1	70.60%
Training	2.7	Equal RF	Positive	153.5	3.9	25433.5	74.00%

Training	2.7	Equal RF	Negative	160.4	5.6	2766.8	58.40%
Training	8.0	Equal RF	Positive	167.4	7	9549.1	57.50%
Training	8.0	Equal RF	Negative	266.4	8.5	6070.1	55.80%
Training	10.0	Equal RF	Positive	318.3	9.3	14795.8	52.80%
Training	10.0	Equal RF	Negative	264.6	6.3	14198.9	58.80%

Table S6. All evaluation metrics. Mean. median and maximum error factor as well as the percentage of datapoints with a prediction error lower than a factor of 10 for all tested models: LC/MS features, Equal RF, closest eluting standard and 2D structural features[11] at each pH and ionization mode for the test set. Green color indicates better performance.

**Dataset**	**pH**	**model**	**Mode**	**Mean**	**Median**	**Max**	**% less than 10**
Test	2.7	LC/MS features	Positive	5.8	2.2	34.8	83.00%
Test	2.7	LC/MS features	Negative	16.9	13.2	50.3	41.70%
Test	8.0	LC/MS features	Positive	12.4	3.3	126.6	79.30%
Test	8.0	LC/MS features	Negative	2.5	1.9	9.3	100%
Test	10.0	LC/MS features	Positive	15.6	3.5	142.3	86.70%
Test	10.0	LC/MS features	Negative	4.4	2.2	23.9	87.90%
Test	2.7	2D structural features	Positive	28.3	3.2	1205.4	84.30%
Test	2.7	2D structural features	Negative	11.5	10.9	23.3	45.80%
Test	8.0	2D structural features	Positive	10.7	5	64.1	67.50%
Test	8.0	2D structural features	Negative	4.7	2.3	26.1	87.50%
Test	10.0	2D structural features	Positive	15.2	5.3	161.9	71.60%
Test	10.0	2D structural features	Negative	10.7	6.5	63.3	66.70%
Test	2.7	Equal RF	Positive	49.3	6	2504.9	60.40%
Test	2.7	Equal RF	Negative	22.3	20.6	142.9	40.00%
Test	8.0	Equal RF	Positive	29.7	11.7	1601.4	54.80%

Test	8.0	Equal RF	Negative	8.4	2.2	88.4	77.60%
Test	10.0	Equal RF	Positive	20.6	12.5	222.5	55.10%
Test	10.0	Equal RF	Negative	11.6	7.1	66.7	58.30%
Test	2.7	Closest eluting standard	Positive	1253.4	11.5	20831.2	45.50%

Table S7. The importance of the features, calculated from the importance function in RRF package, for each model as well as their hyperparameters. Higher values shows higher importance.

Feature	Positive mode pH 2.7	Negative mode pH 2.7	Positive mode pH 8	Negative mode pH 8	Positive mode pH 10	Negative mode pH 10	Mean
m/z	6.66	14.85	7.91	3.53	9.38	8.92	8.54
Odd mass	0.25	0.27	0.5	0	0.57	0.29	0.31
$t_R^{pH\ 2.7}$	2.83	2.62	8.12	1.61	6.5	2.15	3.97
$t_R^{pH\ 8}$	2.53	0	4.13	2.82	6.17	0	2.61
$t_R^{pH\ 10}$	2.72	2.4	3.7	3.12	4.65	7.81	4.07
Negative mode detection	0.44	0	0	0	1.81	0	0.38
$t_R^{pH\ 8}$ - $t_R^{pH\ 2.7}$	6.25	2.87	11.35	3.28	14.28	0	6.33
$t_R^{pH\ 8}$ - $t_R^{pH\ 10}$	4.49	2.64	4.81	1.29	5.32	2.43	3.5
$Int^{+}_{pH\ 2.7}$ / $Int^{-}_{pH\ 2.7}$	6.65	12.4	10.63	7.87	9.52	18.29	10.89
$Int^{+}_{pH\ 8}$ / $Int^{-}_{pH\ 8}$	11.51	1.85	13.82	17.4	13.14	0	9.62
$Int^{+}_{pH\ 10}$ / $Int^{-}_{pH\ 10}$	16.93	2.62	19.01	14.07	20.65	5.5	13.13
Sodium adduct formation	0.5	0	0.72	0.29	2.18	0	0.61

Table S8. All evaluation metrics. Mean. median and maximum error factor as well as the percentage of datapoints with a prediction error lower than a factor of 10 for all tested models: LC/MS features, Equal RF, closest eluting standard and 2D structural features[11] at each pH and ionization mode for the validation set. Green color indicates better performance.

Dataset	pH	model	Mode	Mean	Median	Max	% less than 10
Validation	2.7	LC/MS features	Positive	3.7	2	47.1	93.70%
Validation	2.7	LC/MS features	Negative	12.8	6.6	97.5	64.90%
Validation	8.0	LC/MS features	Positive	5.3	2.4	78.4	93.10%
Validation	8.0	LC/MS features	Negative	7.3	2.7	101.7	83.10%
Validation	10.0	LC/MS features	Positive	7.5	2.9	113.7	86.80%
Validation	10.0	LC/MS features	Negative	14.8	2.5	66.7	62.20%
Validation	2.7	2D structural features	Positive	18.7	3.2	808.7	80.90%
Validation	2.7	2D structural features	Negative	36.5	14.2	414	37.50%
Validation	8.0	2D structural features	Positive	34.3	5.1	1189.1	70.30%
Validation	8.0	2D structural features	Negative	8	3.3	83.2	77.60%
Validation	10.0	2D structural features	Positive	22.2	3.7	619.3	77.20%
Validation	10.0	2D structural features	Negative	5	2.8	37	95.00%

Error propagation

For multiplication, the error of the new variable can be calculated as follows:

If $Q = a \cdot b$

Then $\frac{u(Q)}{Q} = \sqrt{\left(\frac{u(a)}{a}\right)^2 + \left(\frac{u(b)}{b}\right)^2}$

where a and b are measurements and u(a) and u(b) are the uncertainties associated with the same measurements.

Given this relationship the largest error will always dominate. For instance, if the relative uncertainty in variable a is 10x and the relative uncertainty in variable b is 50x then the relative uncertainty in Q ≈ 51.
